# Discovery, characterization and functional improvement of kumamonamide as a novel plant growth inhibitor that disturbs plant microtubules

**DOI:** 10.1038/s41598-021-85501-1

**Published:** 2021-03-23

**Authors:** Takashi Ishida, Haruna Yoshimura, Masatsugu Takekawa, Takumi Higaki, Takashi Ideue, Masaki Hatano, Masayuki Igarashi, Tokio Tani, Shinichiro Sawa, Hayato Ishikawa

**Affiliations:** 1 International Research Organization for Advanced Science and Technology (IROAST), Kumamoto University, Kurokami 2-39-1, Kumamoto, 860-8555 Japan; 2 Graduate School of Science and Technology, Kumamoto University, Kurokami 2-39-1, Kumamoto, 860-8555 Japan; 3 Faculty of Advanced Science and Technology, Kumamoto University, Kumamoto, Japan; 4 Institute of Microbial Chemistry, Tokyo, Japan; 5 Present Address: Graduate School of Pharmaceutical Sciences, Chiba University, Chiba, 263-8522 Japan

**Keywords:** Organic chemistry, Plant cell biology, Plant molecular biology, Natural products, Cytoskeleton

## Abstract

The discovery and useful application of natural products can help improve human life. Chemicals that inhibit plant growth are broadly utilized as herbicides to control weeds. As various types of herbicides are required, the identification of compounds with novel modes of action is desirable. In the present study, we discovered a novel *N*-alkoxypyrrole compound, kumamonamide from *Streptomyces werraensis* MK493-CF1 and established a total synthesis procedure. Resulted in the bioactivity assays, we found that kumamonamic acid, a synthetic intermediate of kumamonamide, is a potential plant growth inhibitor. Further, we developed various derivatives of kumamonamic acid, including a kumamonamic acid nonyloxy derivative (KAND), which displayed high herbicidal activity without adverse effects on HeLa cell growth. We also detected that kumamonamic acid derivatives disturb plant microtubules; and additionally, that KAND affected actin filaments and induced cell death. These multifaceted effects differ from those of known microtubule inhibitors, suggesting a novel mode of action of kumamonamic acid, which represents an important lead for the development of new herbicides.

## Introduction

Discovery and practical application of useful natural products and their derivatives are a means of improving the quality of human life. Secondary metabolites produced by microorganisms, plants and insects, have led to significant advances in medicine and agriculture. Numerous antibiotics and antileukemic agents have been developed from natural products. In addition, diverse types of pesticides, fungicides and herbicides have been sourced from such natural products for use in agriculture. In particular, herbicides that suppress the growth of undesired weeds are essential agents for increasing the yield of crops in modern agriculture, and various types of compounds are used commercially. Several cellular processes in plants such as photosynthesis, amino acid metabolism, cell wall synthesis, mitotic regulation, phytohormone signaling or protein synthesis, have been recognized as some of the typical targets of herbicides^1^. Compounds that inhibit microtubule function are a common class of herbicides that affect the growth of plants by affecting mitotic regulation^2^.

Microtubules are a component of the cytoskeleton and are widely conserved in eukaryotic cells. Tubulin heterodimers, consisting of α-tubulin and β-tubulin form a linear microtubule protofilament and 13 protofilaments form a cylinder-like structure. Microtubules play versatile roles in plant cells, including the determination of cell shapes, cell division and intracellular trafficking^3,4^. Plant cells contain microtubules beneath the plasma membrane at interphase, and these so-called cortical microtubules are thought to direct the organization of cellulose microfibrils via the regulation of cellulose synthase complexes^4,5^. Cortical microtubules of root epidermal cells present in the rapidly elongating region of the root tip are aligned transversely, and cellulose microfibrils follow these microtubules and restrict the direction of the cell expansion, thereby promoting anisotropic cell elongation. Therefore, microtubule function is tightly linked to the morphology of plants^3^. Amino acid substitutions in tubulin-encoding genes induced an oblique cortical microtubule array and left- or right-handed twisting growth in *Arabidopsis thaliana*^6,7^. Similarly, mutations in microtubule-related proteins that regulate microtubule dynamics also caused skewed root growth^8–13^. Moreover, treatment with microtubule-disrupting herbicides (e.g. propyzamide, also known as pronamide), also induced left-handed, skewed root growth^14^. These findings suggest the precise regulation of microtubule function is crucial for the determination of growth direction in plants.

Various types of microtubule inhibitors have been discovered and these agents have contributed substantially to both cytoskeleton research and to agriculture and medicine^2^. In particular, oryzalin, a dinitroaniline-class compound, propyzamide, and benzamide-related compounds and their analogues, are all capable of inhibiting microtubule function, and in turn, of inhibiting the growth of plants; hence, their widespread use as herbicides. However, because microtubules are a fundamental component of both plant and animal cells, most microtubule inhibitors produce cytotoxicity of both types of cells. Therefore, a limited number of anti-microtubule agents have been used for practical purposes, despite their recognized suitability as herbicides.

*Streptomyces* is a genus consisting of the family Streptomycetaceae includes aerobic, Gram-positive, filamentous bacteria and is well-recognized for its ability to produce diverse secondary metabolites. Thus, it is regarded as one of the most important sources of new biologically active natural products. In the current studies, we discovered a novel compound named kumamonamide, which we isolated from *Streptomyces werraensis* MK493-CF1 and *S. werraensis* ISP 5486. The structure of kumamonamide was characterized and its unique *N*-alkoxy pyrrole skeleton was determined by spectroscopic analysis and total synthesis. Kumamonamic acid, a synthetic intermediate of kumamonamide and its derivatives were found to inhibit growth and germination of the popular plant model, *Arabidopsis thaliana*. In a structure–activity relationship study, we discovered that a compound with C9 modified to kumamonamic acid, named as kumamonamic acid nonyloxy derivative (KAND), remarkably increased the inhibitory effect on growth and germination. Notably, the newly discovered plant-growth inhibitors also affected the growth of tobacco and liverwort, and did not produce cytotoxicity of bacteria or HeLa cells. Furthermore, several kumamonamic acid derivatives induced twisting-root phenotypes, implying these derivatives would target microtubules, either directly or indirectly. Consistent with this idea, our observations of immunohistochemically labeled or fluorescent protein-labeled microtubules revealed that treatment with KAND depolymerized microtubules. Moreover, treatment with kumamonamic acid derivatives disrupted actin microfilaments. In summary, we have discovered a novel, plant-specific growth inhibitor with a unique mode of action involving disruption of the cytoskeleton.

## Results

### Discovery and total synthesis of kumamonamide

The strain MK493-CF1 was isolated from soil in Shinagawa-ku, Tokyo. Strain MK493-CF1 formed well-branched substrate mycelia. The partial 16S ribosomal RNA gene sequence (1422 bp) was determined. The strain showed high similarity with *S. werraensis* (NBRC 13404^T^ = ISP 5486, 1421/1422 bp, T: Type strain, 99.93%). From this result, this strain was determined to be the closely related to the type strain of *S. werraensis*. Therefore, we tentatively designated this strain as *S. werraensis* MK493-CF1. *S. werraensis* ISP 5486^T^ also produced same bioactive compound. As earlier studies to obtain natural products from this microorganism are rare, further chemical investigations were conducted. After culturing *S. werraensis* MK493-CF1 on barley media by solid-state fermentation for 14 days at 30 °C, the cultured media were extracted with 50% EtOH. A 60 mL sample was dried and 59.5 mg of the crude extract was obtained. The crude extract was subjected to reverse-phase HPLC, yielding *N*-methoxy-1H-pyrrole-2-carboxamide (**1**, named as kumamonamide, 36.0 mg). The total amount of **1** was ~ 60% of the crude extract. Therefore, we decided to study the detailed characteristics of kumamonamide **1**.

**Figure 1:**
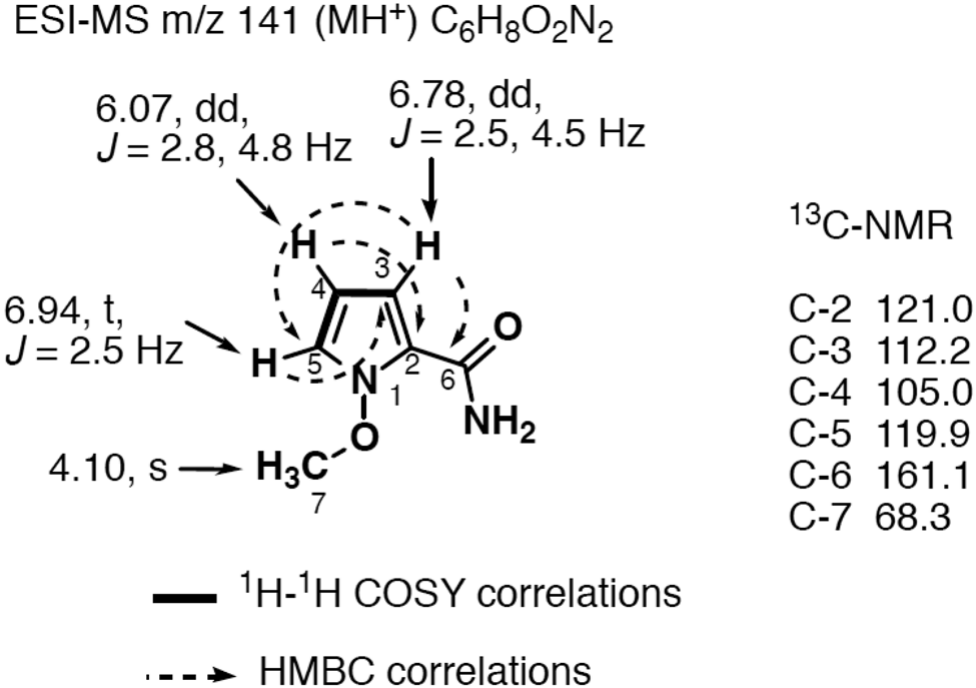
Structure of kumamonamide and its NMR data.

Kumamonamide **1** was obtained as a white amorphous powder, and high-resolution mass spectrometry (HRESIMS) supported C_6_H_8_N_2_O_2_ (Fig. 1). The C2-substituted pyrrole moiety of the compound was determined by the presence of three characteristic aromatic protons at δ_H_ 6.94 (1H, t, *J* = 2.8, 4.8 Hz, H-4), δ_H_ 6.78 (1H, d, *J* = 2.5, 4.5 Hz, H-5), and δ_H_ 6.78 (1H, d, *J* = 2.5 Hz, H-6) in a ^1^H-NMR spectrum, while the ^13^C-NMR spectrum revealed the presence of four *sp*^2^ carbons. The presence of an amide group at the C2 position was estimated by HMBC correlations from the C-3 proton to the amide carbonyl carbons at δ_C_ 161.1. Further, ^1^H- and ^13^C-NMR peaks at δ_H_ 4.10 (3H, S) and δ_C_ 68.3 indicated an *N*-methoxy group in the molecule. Although the correct location of the methoxy group was not determined by spectroscopic analysis such as Nuclear Overhauser enhancement difference spectra” and abbreviation (NOEDF), *N*-methoxy-1*H*-pyrrole-2-carboxamide became the first candidate compound.

**Figure 2:**
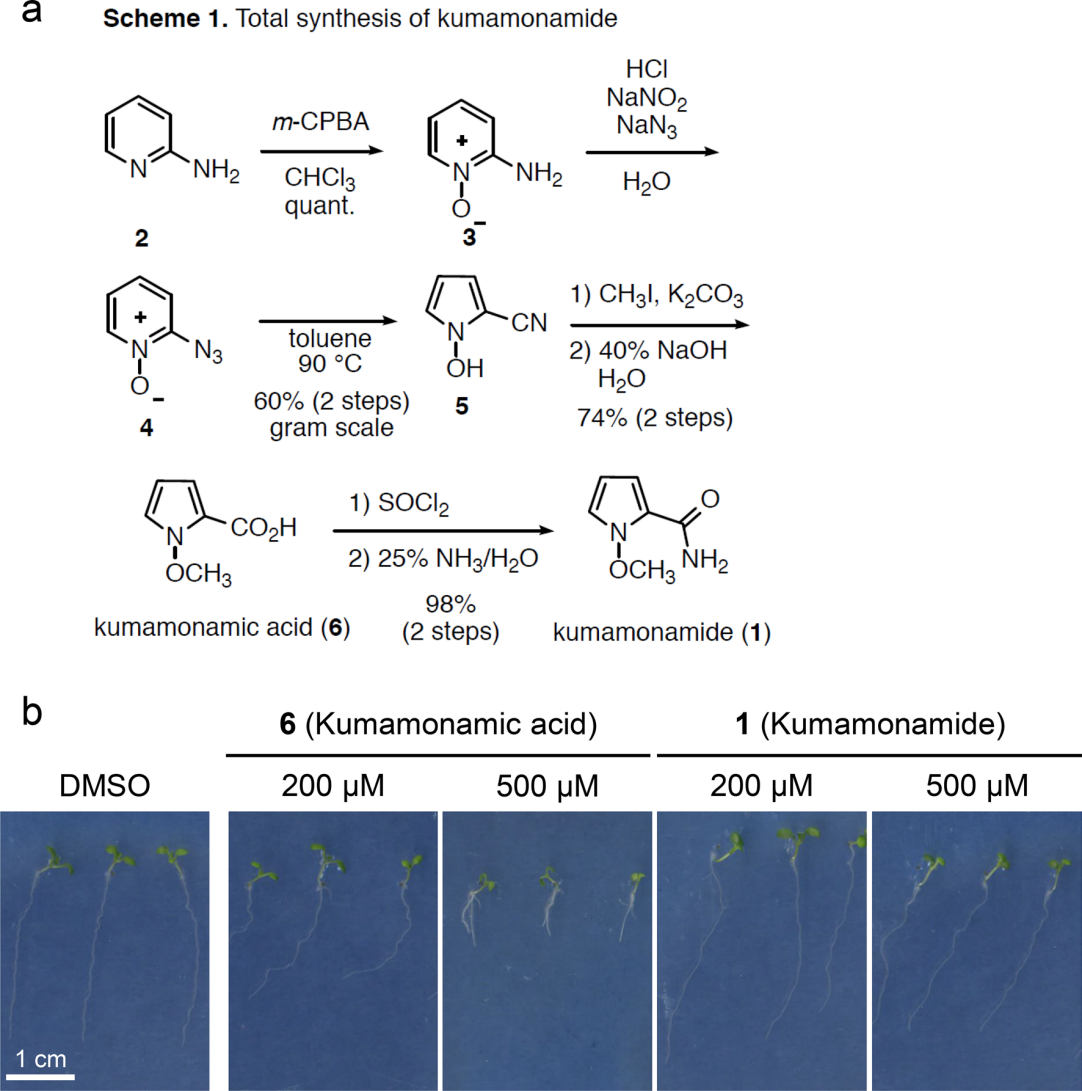
Total synthesis and bioactivity analyses of kumamonamide and kumamonamic acid. (**a**) Total synthesis of kumamonamide. (**b**) Seven-day-old, wild-type *Arabidopsis* Columbia (Col) seedlings were grown on Murashige and Skoog (MS) plates with the indicated concentrations of kumamonamic acid **6** or kumamonamide **1**. Scale bar = 1 cm.

To determine the correct structure of **1**, total synthesis was conducted (Fig. 2a). Commercially available 2-amino pyridine **2** was treated with *m*-CPBA to provide the corresponding *N*-oxide **3** in quantitative yield. After azidation of the 2-amino group of **2**, the ring contraction reaction reported by Abramovitch in benzene at 90 °C was carried out to provide gram quantities of the desired 1-hydroxy-1H-pyrrole-2-carbonitrile **5** in 60% yield (two steps)^15,16^. Next, methylation followed by hydrolysis of **4** produced 1-methoxy-1H-pyrrole-2-carboxylic acid (named as “kumamonamic acid”, **6**) in good yield (70%, two steps). Finally, amidation using aqueous ammonia solution via an acyl chloride intermediate of **6** provided kumamonamide **1** in 98% yield. All spectroscopic data of synthetic **1** were similar to those for isolated **1**; thus, the structure of **1** was determined.

### Bioactivity of kumamonamide and kumamonamic acid

**Figure 3:**
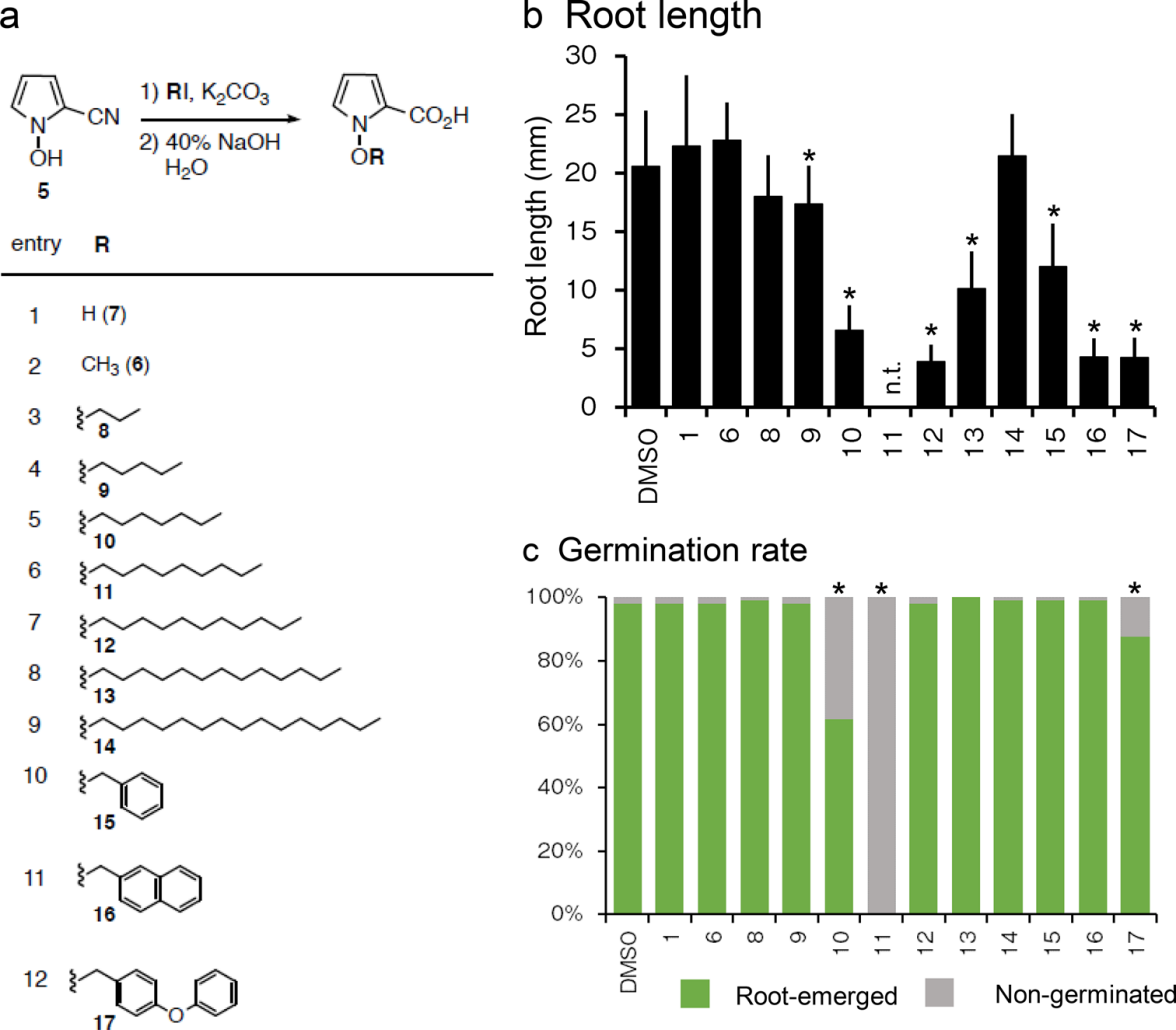
Structure–activity relationship study of kumamonamide and its related compounds. (**a**) The structure of and synthetic protocols for analogues. (**b**) Quantification of the root lengths of the 7-day-old seedlings grown on MS media with or without 50 μM kumamonamide derivatives. Asterisks indicate significant differences from mock treatment (*t*-tests, p < 0.05). n > 18. Data are shown as mean ± SD. n.t. indicates not tested due to more than 50% of seeds were not germinated. (**c**) Quantification of the germination rate of the 7-day-treated seeds incubated on MS media with or without 200 μM kumamonamide and its related compounds. Asterisks indicate significant differences from mock treatment (chi-square tests). n = 96.

First, we assessed the bioactivity of kumamonamide and an intermediate for their potential to modulate plant growth. We added kumamonamide **1** or kumamonamic acid **6** to MS agar media at various concentrations and grew *Arabidopsis* seedlings on the media. These assays revealed that a high concentration (500 μM) of **6** inhibited root growth (Fig. 2b). Next, we produced various derivatives by replacing the N1 position of **6** and subjected these to a structure–activity relationship study (synthesis procedure of analogues is described in the Supporting information (SI)). *Arabidopsis* seedlings were grown on media containing 50 μM of kumamonamic acid derivatives and the root length was measured. As shown in Figs. 3a,b and S1, kumamonamic acid derivatives with different length linear alkoxy chains (**9**, **10**, **11**, **12** and **13**) or bulky alkoxy chains (**15**, **16** and **17**) at the N1 position, displayed significant inhibition of root growth. Additionally, we found that application of 200 μM of **10**, **11** or **17** inhibited germination (Figs. 3c and S2).

Interestingly, the addition of alkyl side chains longer than C9 lowered the inhibitory activity, suggesting that a specific size of side chain is required for kumamonamic acid -related compounds to exert their bioactivity.

### Analysis of the inhibitory effect of the kumamonamic acid C9 derivative on plant growth

**Figure 4:**
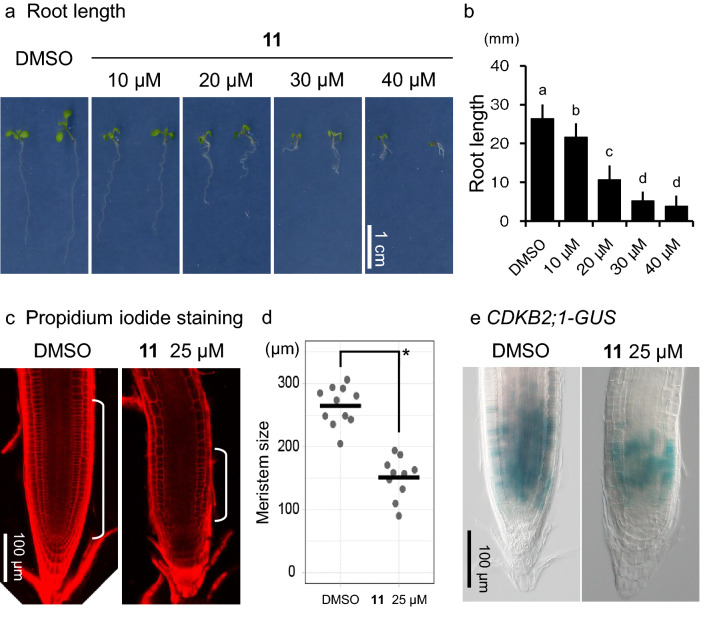
Analysis of the inhibitory effect on growth of the kumamonamic acid derivative, kumamonamic acid nonyloxy derivative (KAND). (**a**) Seven-day-old, wild-type Col seedlings were grown on MS plates with the indicated concentrations of KAND **11**. Scale bar = 1 cm. (**b**) Quantification of the root length. Letters indicate significant differences (Tukey’s HSD test, p < 0.05). n > 16. Data are shown as average ± SD. (**c**) Confocal microscopy of propidium iodide-stained wild-type Col roots grown on MS plates with or without 25 μM KAND **11**. White brackets indicate root meristem. Scale bar = 100 μm. (**d**) Quantification of the root meristem size (n = 10 to 11). Statistical differences were determined by *t*-test (p < 0.05). Bars indicate mean meristem size. (**e**) Differential interference contrast (DIC) microscopy of root meristems containing *CDKB2;1pro:CDKB2;1-GUS* constructs, Five-day-old seedlings grown on MS plates with or without 25 μM KAND were stained and analyzed.

As structure–activity relationship analyses revealed that C9 modified to kumamonamic acid, kumamonamic acid nonyloxy derivative (hereafter KAND **11)**, was the most potent plant growth inhibitor, we characterized KAND **11** in more detail. Treatment of *Arabidopsis* with 50 μM KAND **11** almost totally blocked germination, while lower concentrations (40, 30, 20 or 10 μM) of KAND **11** repressed root growth as dose-dependent manner (Fig. 4a,b). To test whether KAND **11** affected the activity of root meristems, we examined propidium iodide (PI)-stained root meristems and measured the size of meristematic regions. The meristem size of seedlings grown on media containing 25 μM KAND **11** was 151.1 ± 32.5 μm, while that grown on DMSO-containing control media was 264.7 ± 30.8 μm (Fig. 4c,d), suggesting that KAND **11** lowered cell proliferation in the root meristem. Consistent with this, treatment with KAND **11** reduced the number of cell division marker CDKB2;1p::CDKB2;1-GUS signals in the root meristem (Fig. 4e)^17^. These results imply that KAND **11** inhibited root growth via the reduction of cell proliferation activity.

### Kumamonamic acid derivatives produce cytotoxicity in a broad range of plants, but not in other organisms

**Figure 5:**
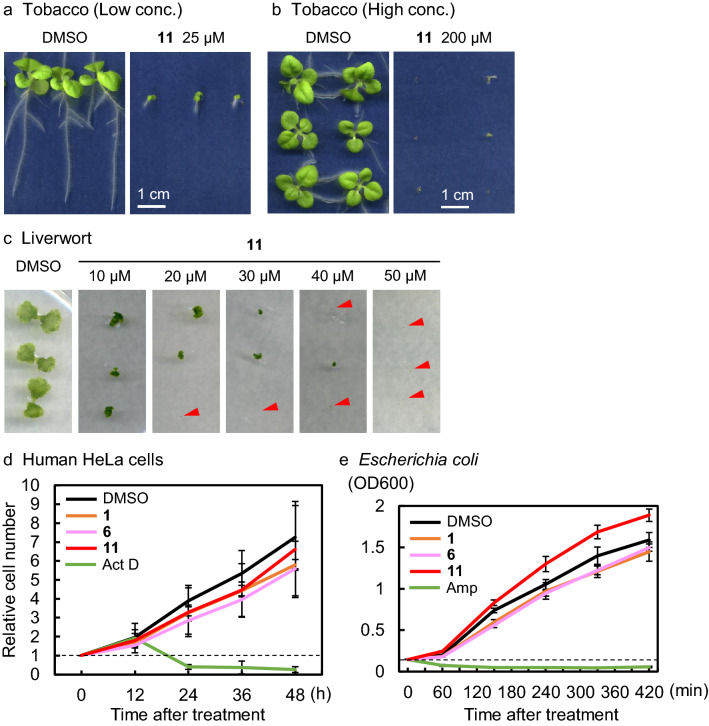
Inhibitory effects on growth of the KAND 11 in non-*Arabidopsis* organisms. (**a**) Two-week-old, wild-type *Nicotiana tabacum* SR-1 seedlings were grown on vertically placed MS plates with 25 μM of KAND **11**. (**b**) Two-week-old, wild-type *N. tabacum* SR-1 seedlings were grown on horizontally placed MS plates with 200 μM of KAND **11**. (**c**) Two-week-old, wild-type *Marchantia polymorpha* Tak-1 gemmae were grown on Gamborg B5 plates with the indicated concentrations of KAND **11**. Red arrows indicate gemmae that ceased growing for the duration of the 2-week incubation. (**d**) Cell proliferation assay of HeLa cells. The numbers of viable cells were measured using a Cell counting kit-8 (Dojindo) at fixed time intervals. As a control, HeLa cells were treated with 5 μg/ml of actinomycin D (Act D), which inhibits transcription by RNA polymerases and causes cell death. The assays were conducted in triplicate. (**e**) Cell proliferation assay of *Escherichia coli* bacteria. Growth of *E. coli* was analyzed by measuring OD600. As a control, cells were treated with 50 μg/ml of ampicillin (Amp), which inhibits bacterial cell wall synthesis. The assays were conducted in triplicate.

The phytotoxicity of KAND **11** was further tested using other eudicot plants, tobacco (*Nicotiana tabacum*), and a model organism for basal land plants, liverwort (*Marchantia polymorpha*). Similar to observations with *Arabidopsis*, tobacco SR-1 seedlings grown on media containing 25 μM KAND **11** produced shorter roots (Fig. 5a). Further, 40 out of 48 seeds were germinated on the 200 μM KAND **11**-containing plates while all 48 seeds were germinated on the mock-treated media, indicating that the higher concentration of KAND significantly (p < 0.05; chi-squared test) inhibited the germination of tobacco (Fig. 5b). Moreover, KAND **11** inhibited the growth of thallus in liverwort at similar concentrations to those effective in *Arabidopsis* (Fig. 5c). These results revealed that KAND **11** is capable of inhibiting the growth of a broad spectrum of plants. We then examined the possible cytotoxicity of kumamonamide-related compounds in other organisms, namely human HeLa cells and *Escherichia coli* DH5α strain, as representatives of higher animal cells and bacteria, respectively. In a series of cell proliferation assays, we observed that kumamonamide **1**, kumamonamic acid **6** and KAND **11** did not affect the growth of HeLa cells or *Escherichia coli* at the concentration of 100 μM (Fig. 5d,e).

### Evidence that kumamonamic acid derivatives affect microtubules

**Figure 6:**
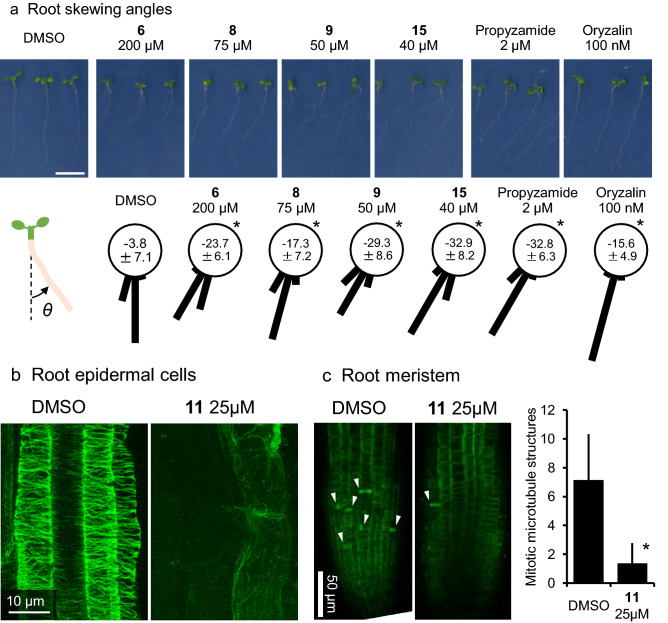
Kumamonamic acid and its derivatives altered cortical microtubules in *Arabidopsis*. (**a**) Root skewing angles measured in the presence of the indicated concentrations of different kumamonamic acid derivatives. The effects of two compounds, propyzamide and oryzalin, known to inhibit microtubules, were also analyzed. The illustration indicates the criteria used for the measurement of root growing angles. Asterisks indicate significant differences from mock treatment (*t*-tests, p < .0.05). n > 19. Scale bar = 1 cm. (**b**) Cortical microtubules in epidermal cells of the elongation zone. Microtubules of wild-type *Arabidopsis* Col roots grown on MS plates with or without 25 μM KAND **11** were visualized by immunohistochemical staining using a β-tubulin primary antibody and an Alexa Fluor-conjugated secondary antibody. Scale bar = 10 μm. (**c**) Mitotic microtubule structures in the root meristem. Microtubules were visualized by immunohistochemical staining. Mitotic structures including preprophase bands, spindles and phragmoplasts in the confocal images were counted. Arrowheads indicate mitotic microtubule structures. Asterisks indicate significant differences from mock treatment (*t*-tests, p <  0.05). n > 9. Scale bar = 50 µm.

In order to decipher the mode of action of the cytotoxicity produced by the kumamonamide-related compounds, we reanalyzed kumamonamic acid derivatives that exert moderate inhibitory effects. As shown in Figs. 2b, 6a, seedlings grown on agar plates containing a high concentration (200 μM) of kumamonamic acid **6** produced shorter and left-skewed roots (θ = – 23.7 ± 6.1), while those grown on control media produced almost straight roots (θ = – 3.8 ± 7.1). This characteristic skewing growth is known to arise from the dysfunction of cortical microtubules^14,18^. Consistent with this conclusion, the microtubule-destabilizing drugs, propyzamide and oryzalin, induced similar root-skewing under our growth conditions (Figs. 2b, 6a). Simultaneously, we tested the kumamonamic acid derivatives and selected several of them at specific concentrations that induce skewing root growth. Compounds **8**, **9** and **15** altered the direction of root growth at 75 μM, 50 μM and 40 μM, respectively, suggesting that the compounds are potent to destabilize microtubules (Figs. 2b, 6a). We also examined the most potent kumamonamic acid derivative, KAND **11**, at a lower concentration (15 μM) and found that the administration of KAND **11** inhibited root growth and their growth directions were being ununiformed though they tend to show left-directed skewing (Fig. S3). Because higher concentrations of microtubule-destabilizing drugs occasionally inhibited the growth of plants rather than induce root skewing, we then assessed the possibility that KAND **11** affects microtubules by observing the cortical microtubules in root epidermal cells. Immunohistochemistry using an anti-β-tubulin antibody in root epidermal cells of seedlings treated with 25 μM KAND **11** revealed that almost all cortical microtubules in the epidermal cells of elongation zone had disappeared (Fig. 6b). These results indicate that kumamonamic acid and its derivatives target microtubules to disrupt them, either directly or indirectly, and that these compounds are novel microtubule inhibitors.

Although kumamonamic acid is capable of disturbing microtubule function, its mode of action is predicted to be different from canonical microtubule depolymerizing agents. For example, a higher concentration of microtubule depolymerizing agents, e.g. propyzamide and oryzalin, induce anisotropic expansion of epidermal cells whereas KAND **11** did not^19^. Further, the simultaneous administration of KAND **11** and propyzamide resulted combined responses in the root growth; both propyzamide-induced twisting growth and KAND **11**-induced growth inhibition was observed (Fig. S4). We also analyzed the response of *propyzamide hypersensitive 1–1 *(*phs1-1*) mutants to the KAND **11**. *phs1-1* harbors a point mutation in an atypical tubulin kinase and produce shorter roots when treated by propyzamide^9,20^. *phs1-1* mutant seedlings grown on agar medium containing KAND **11** exhibited shorter roots, similar to that grown on propyzamide (Fig. S5).

Additionally, we observed mitotic microtubule structures, e.g. preprophase band, spindle and phragmoplast, in the root meristem of KAND **11** treated seedlings. In consistent with the observations with CDKB2;1p::CDKB2;1-GUS, the number of mitotic microtubules observed was significantly reduced (Fig. 6c).

### Kumamonamic acid and its derivatives affect microtubules and actin filaments

**Figure 7:**
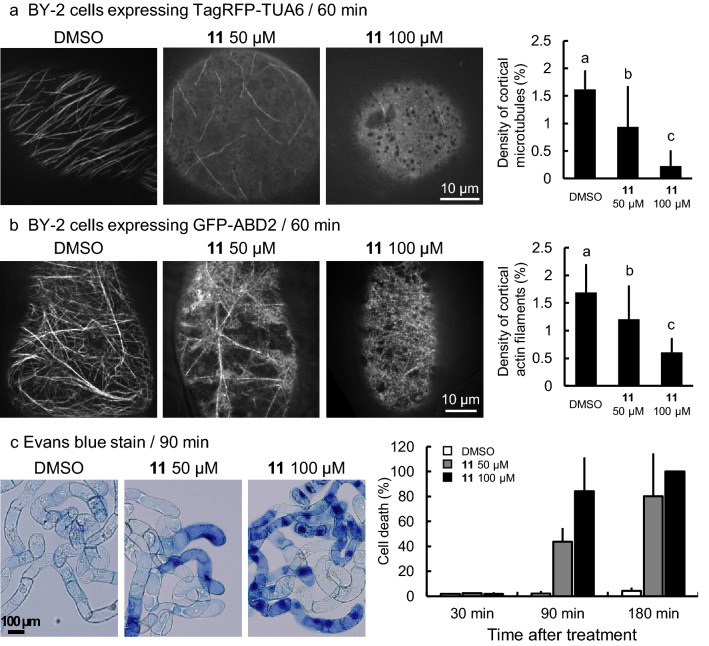
KAND affected cortical microtubules, actin filaments and cell viability in tobacco BY-2 cells. (**a**) Cortical microtubules in BY-2 cells visualized by the presence of TagRFP-TUA6. BY-2 cells treated with KAND **11** (50 μM or 100 μM) or DMSO, were examined using confocal microscopy. The density of cortical microtubules was calculated from micrographs acquired from 25 independent cells. Letters indicate significant differences (Tukey’s HSD test, p < 0.05). Scale bar = 10 μm. (**b**) Cortical actin filaments in BY-2 cells visualized by the presence of GFP-ABD2. BY-2 cells treated with KAND **11** (50 μM or 100 μM) or DMSO were examined using confocal microscopy. The density of cortical actin filaments was calculated from micrographs acquired from 25 independent cells. Letters indicate significant differences (Tukey’s HSD test, p < 0.05). Scale bar = 10 μm. (**c**) Dead BY-2 cells visualized by Evans blue staining. BY-2 cells treated with KAND **11** (50 μM or 100 μM) or DMSO were examined by bright field microscopy. n = 3. Scale bar = 100 μm.

To characterize the cytotoxicity of KAND **11** with subcellular resolution, we treated tobacco BY-2 suspension cells with KAND **11** and observed their response. First, we added KAND **11** to BY-2 cells expressing TagRFP-TUA6 that fluorescently labels microtubules to evaluate the impact of KAND **11** on cortical microtubules. The densities of cortical microtubules were evaluated by the image analyses that quantify the percentage of pixels for the cytoskeleton in the pixels for cytoplasm. Resulted in the analyses, the densities were significantly decreased to 0.94 ± 0.74% or 0.23 ± 0.28% by treatment with 50 μM or 100 μM KAND **11** for 1 h, respectively, while the density in DMSO-treated cells was 1.61 ± 0.34% (Fig. 7a). These results are consistent with the observations in *Arabidopsis* that KAND **11** treatment induced depolymerization of cortical microtubules (Fig. 6b). We also examined a BY-2 line with GFP-ABD-labeled actin filaments after treatment with the same concentration of KAND **11** and observed that KAND **11** treatment disrupted actin filaments. The densities of actin filaments were significantly decreased to 1.20 ± 0.62% or 0.61 ± 0.26% by treatment with 50 μM or 100 μM KAND **11** for 1 h, respectively, while the density in DMSO-treated cells was 1.69 ± 0.51% (Fig. 7b). These results are in contrast to the effects of propyzamide which did not affect actin filaments and the effects of latrunculin B, an actin-depolymerizing agent, which did not affect microtubules either (SI Fig. S6). Besides, treatment of kumamonamide **1**, kumamonamic acid **6** or KAND 11 did not affect microtubules in HeLa cells (SI Fig. S7). Therefore, the mode of action of KAND **11** is considered to be different to that of known cytoskeleton-disrupting agents. Furthermore, our microscopic observations of KAND **11** -treated BY-2 cells identified the onset of cell death during KAND **11** treatment and revealed that the ratio of Evans blue-stained dead cells were not obviously increased by 30 min KAND **11** treatment while the ratio of dead cells were increased to 43.7% or 80.1% by the treatment of 50 µM or 100 µM KAND for 90 min, respectively (Fig. 7c). Taken together, these data indicate that the novel kumamonamic acid derivative, KAND **11,** is a plant-specific cytoskeleton inhibitor with a previously uncharacterized mode of action.

## Discussion

The discovery and application of novel natural products has led to significant advances in various aspects of human life including medicine or agriculture. Historical studies have been paid to obtain useful compounds from resources in nature. In particular, actinomycetes have been an excellent bioresource due to their capacity to produce various secondary metabolites such as avermectin, a lead compound for ivermectin, which is used as an antiparasitic nematode antibiotic; and bleomycin and its derivatives, used in medicine as anticancer agents^21,22^. Similarly, various herbicidal compounds have been identified from actinomycetes and some have been used commercially^1,23^. Thus, analyzing metabolites of actinomycetes with the goal of isolating natural products with desirable bioactivity has been recognized as an effective strategy. In the present study, we discovered a novel compound, kumamonamide, from *S. werraensis* and succeeded in a total synthesis. Kumamonamic acid, a synthetic intermediate of kumamonamide and its derivatives induced characteristic twisting root and displayed moderate to strong herbicidal activities, and disrupted the plant microtubules, either directly or indirectly,. However, the mode of action of kumamonamic acid is likely to be different from existing microtubule inhibitors, as KAND **11** also disrupted actin filaments and produced cell death, suggesting that kumamonamic acid and its derivatives affect regulatory mechanisms of a broad range of cytoskeleton structures.

Further detailed characterization of kumamonamic acid will help to better understand the mode of action of kumamonamic acid. In particular, assessing the binding ability of kumamonamic acid to the reconstituted microtubules is the next task to discriminate whether kumamonamic acid and its derivatives directly target and depolymerize microtubules or the destabilization of microtubules is the downstream consequences of its actions. Moreover, identification of the location of action of kumamonamic acid to the plant cells and the molecular target, in case of microtubules are not the direct target, will provide further understandings of the characteristics of related compounds and possible ways to improve the herbicidal activity. Our bioactivity assay revealed a unique cytotoxic ability of kumamonamic acid toward the growth of plants including *Arabidopsis*, tobacco, and liverwort, whereas neither *E. coli* nor HeLa cells were affected. Little or no toxicity toward animal cells is an advantageous characteristic of kumamonamic acid derivatives, if it is to be developed as a herbicide used in open, agricultural fields. Indeed, because microtubules are a common structure of eukaryotes, their selective inhibition in plants is a critical requirement for herbicides. For example, propyzamide, a microtubule-depolymerizing agent that binds directly to tubulin and inhibits polymerization is used as a herbicide due to its low toxicity in animal cells^24^. Unlike propyzamide, related benzamide-class compounds display divergent target specificities. RH-4032 or zoxamide inhibit microtubules of animal cells or oomycetes, respectively, in addition to plant microtubules, and zarilamide has been used as a fungicide because of its low phytotoxicity^25–27^. The newly discovered kumamonamic acid and its derivatives exert selective cytotoxicity against plants, but notably, further modifications may alter their target specificity, and thence perhaps provide additional derivatives that are employed to control pathogenic fungi or oomycetes.

The unique characteristics of kumamonamic acid and its derivatives are useful for both their development as a herbicide and for their use as research tools. The importance of the cytoskeleton in the control of cell shapes in plants has been widely recognized. Earlier studies identified that plants have evolved sophisticated mechanisms to organize cortical microtubules via the control of microtubule dynamics to correctly direct morphogenesis. A large number of molecules responsible for the regulation of microtubule activity have been identified, and related studies are still ongoing^3,4,28^. Our current understanding of microtubule dynamics in plant cells does not completely explain the mechanisms underlying cortical microtubule organization. For example, although both propyzamide and oryzalin can depolymerize microtubules, propyzamide induced major twisting of the roots, while oryzalin produced a relatively mild effect. Furthermore, mutations in tubulin that stabilize microtubules, also induced right-handed skewing of the root, whereas taxol, which also stabilizes microtubule dynamics, did not^7^. Therefore, the investigation and identification of the molecular targets of kumamonamic acid should provide new insights into the regulation of cortical microtubules in plants. Similarly, future comparison between chemicals that effectively induce skewing growth, e.g. propyzamide, and that are less effective, e.g. oryzalin or kumamonamic acid, would provide hints for the understandings that how the twisting growth earn.

On the other hand, defense-related rearrangement of cytoskeleton is another possibility to explain the cytotoxicity of kumamonamic acid. Infection of pathogens or administration of elicitors to plant cells occasionally triggers disruption of cytoskeleton and subsequent cell death^29^. For instance, cryptogein obtained from oomycete, has been reported to disrupt microtubules and actin filaments prior to cell death in tobacco cells, similar to the case of KAND treatment^30,31^. The resemblance between the defense responses and kumamonamic acid-induced cellular responses let us hypothesize that they trigger a common cellular process though the rapid and strong effect of kumamonamic acid than that of cryptogein is noticeable. However, studies have revealed that the disruption of actin filaments promotes spontaneous cell death whereas the disruption of microtubules is not always accompanied^29^. Moreover, whether neither the pathogens nor elicitors induce twisting growth of roots as kumamonamic acid derivatives did are yet to be studied. Owing to this, the molecular insights which link the defense response and cytoskeleton are attractive questions to be solved. By taking advantage of kumamonamic acid-related compounds that they are low-molecular-weight compounds and the presence of a series of derivatives with various strengths, they may let to an opportunity to tackle the unidentified cellular mechanisms.

In summary, the discovery and application of novel compounds that modulate microtubule dynamics will provide a powerful technique for resolving the complicated molecular mechanisms underlying the determination of plant cell shapes. In this context, the newly developed compound, kumamonamic acid, which affects microtubules and actin filaments, and can induce cell death, may provide an opportunity to decipher the link between the control of microtubules and these other mechanisms. Together, chemical and biological analyses using kumamonamic acid will assist our understanding of the molecular regulatory mechanisms controlling the cytoskeleton in plants.

## Methods

### Fermentation and purification of kumamonamide from actinomycetes

*S. werraensis* MK493-CF1 was inoculated into a 500-mL baffled Erlenmeyer flask containing 110 mL of a seed medium consisting of 2% (w/v) galactose, 2% (w/v) dextrin, 1% (w/v) Bacto-soytone (Thermo Fisher Scientific, Inc.), 0.5% (w/v) corn steep liquor (KOGOSTCH Co., Ltd., Japan), 0.2% (w/v) (NH_4_)_2_SO_4_ and 0.2% CaCO_3_ in deionized water (pH 7.4 before sterilization). The seed culture was incubated in a rotary shaker (180 r.p.m.) at 27 °C for 2 days. The producing culture was carried out by solid state fermentation. Seed culture (7 mL) was transferred into a 500 mL K-1 flask containing 40 g production medium consisting of pressed barley 15 g (MUSO Co., ltd., Japan) and deionized water 25 g (pH was not adjusted before sterilization). The fermentation was carried out at 30 °C for 14 days in the dark condition. The fermentation material was extracted with 40 mL/flask of EtOH and centrifuged (1500×*g*, 4 °C, 10 min.). This culture supernatant (60 mL) was extracted with 10% MeOH/EtOAc. The organic layer was evaporated under reduced pressure to produce a residue (59.5 mg), which was subjected to HPLC over a reversed-phase column (SHISEIDO CAPCELL PAK C18 UG120, 5 μm, 10 mm I.D. × 250 mm in length) with gradient elution (0–10 min: 90% H_2_O/CH_3_CN, 10–35 min: 90% H_2_O/CH_3_CN to 70% H_2_O/CH_3_CN (gradient), 35–45 min: 90% H_2_O/EtOH, 45–155 min: 90% H_2_O/EtOH to 100% EtOH (gradient), 155–200 min: 100% EtOH) at a flow rate of 1.5 mL/min to isolate kumamonamide (**1**, 36.0 mg) as a white amorphous powder.

Kumamonamide (**1**); ^1^H-NMR (500 MHz, CDCl_3_) δ 6.93 (t, *J* = 2.5 Hz, 1H), 6.76 (dd, *J* = 4.3, 1.8 Hz 1H), 6.05 (t, *J* = 3.8 Hz, 1H), 4.08 (s, 3H); ^13^C-NMR (125 MHz, CDCl_3_) δ 161.1, 121.0, 119.9, 112.2, 105.0, 68.3; ESI-HRMS [M + H]^+^: calculated for [C_6_H_9_N_2_O_2_]^+^: 141.0659, found: 141.0663; IR ν_max_ 3451, 3414, 3173, 2938, 1603, 1593, 1537 cm^–1^.

### Organisms and growth conditions

Columbia (Col-0) seeds were obtained from Arabidopsis Biological Resource Center (ABRC) with permission for use of academic research. The Col-0 seeds were propagated and maintained in our lab condition and used as the wild-type *Arabidopsis* plant. *Arabidopsis* seeds were surface sterilized and germinated on half-strength Murashige and Skoog medium (Fujifilm Wako Pure Chemical) containing 2% sucrose (Fujifilm Wako Pure Chemical), 0.05% (w/v) 2-(4-morpholino) ethanesulfonic acid (MES) and 1.5% agar (Fujifilm Wako Pure Chemical) at pH 5.7, under continuous light at 23 °C. *phs1-1* mutant seeds were provided by T. Hashimoto (Nara Institute of Science and Technology).

The SR-1 strain seeds were provided by T. Hashimoto (Nara Institute of Science and Technology) and used as the wild-type tobacco plant. Tobacco seeds were surface sterilized and immersed in sterile water for three nights to promote germination, then plated on half-strength Murashige and Skoog medium containing 2% sucrose, 0.05% (w/v) MES and 0.8% gellan gum (Fujifilm Wako Pure Chemical) at pH 5.7, and incubated under continuous light at 23 °C.

The Tak-1 strain was provided by T. Kohchi (Kyoto University) and used as the standard experimental line of *Marchantia polymorpha*. Gemma were obtained from sterilized cultured plants, then plated on Gamborg's B5 Medium (Fujifilm Wako Pure Chemical) containing 1% sucrose and 0.3% gellan gum, and incubated under continuous light at 23 °C.

Tobacco BY-2 (*Nicotiana tabacum* L. cv. Bright Yellow 2) cells were provided by S. Hasezawa (The University of Tokyo). BY-2 cells were diluted 95-fold with modified Linsmaier and Skoog medium supplemented with 2,4-dichlorophenoxyacetic acid at weekly intervals^32^. Cell suspensions were agitated on a rotary shaker at 130 rpm at 27 °C in the dark. The cells were washed with 10 volumes of fresh medium and suspended in the same medium. Transgenic BY-2 cell lines stably expressing a microtubule marker, TagRFP-TUA6, or an actin filament marker, GFP-ABD2, under the cauliflower mosaic virus 35S promoter were established as described^33–35^. These cell lines could be maintained and synchronized by procedures similar to those used for the original BY-2 cell line.

HeLa cells were cultured using Dulbecco’s Modified Eagle Medium (DMEM) (Life Technologies) supplemented with 10% fetal bovine serum, in the presence of 1.2 units/mL penicillin and 1.2 μg/mL streptomycin, at 37 °C in a 5% CO_2_ incubator.

*Escherichia coli* DH5α was cultured with shaking in LB broth at 37 °C.

All experiments described in this manuscript were performed in accordance with guidelines and regulations for biosafety in Japan.

### Herbicidal activity assays

Compounds were dissolved in dimethyl sulfoxide (DMSO; Fujifilm Wako Pure Chemical) as a stock solution and diluted into the MS medium for *Arabidopsis* and Tobacco or the Gamborg's B5 Medium for liverwort. For the root growth inhibition assays, more than 10 seeds per one petri dish were plated on the agar medium containing indicated compounds or DMSO. Seeds were incubated for 7 days in the growth chamber. The seedlings were photographed and the root length were measured. For the germination assays of *Arabidopsis*, 48 seeds per one petri dish were plated on the agar medium containing 200 µM compounds or DMSO. The Arabidopsis seeds were incubated in the growth chamber and the number of germinated seedlings were counted at 7 day after germination (dag). For the germination assays of tobacco, 24 seeds per one petri dish were plated on the agar medium containing 200 µM KAND or DMSO. The tobacco seeds were incubated in the growth chamber and the number of germinated seedlings were counted at 14 dag. For the liverwort growth inhibition assays, 9 gemmae per one petri dish were plated on the agar medium containing indicated concentrations of KAND or DMSO and incubated for 14 days in the growth chamber.

### Microscopic observation of root meristem organization

Root meristem organization was visualized using seedlings stained with 5 mg/mL propidium iodide (PI). PI signals were visualized by fluorescence microscopy using a TCS SPE confocal laser scanning microscope (Leica Microsystems).

### GUS staining and microscopy

Histochemical β-glucuronidase (GUS) staining of roots was performed based on the protocol described by Malamy and Benfey^36^. Seedlings were fixed in 90% acetone overnight, stained in the GUS buffer with 0.5 mg/mL 5-bromo-4-chloro-3-indolyl-β-d-glucuronide for 1 h, mounted in chloral hydrate solution (8 g chloral hydrate, 2 mL water, and 1 mL glycerol), and observed by differential interference contrast microscopy, using an Axio Imager M1 microscope (Carl Zeiss).

### Analyses of root skewing angles

Seven-day-old seedlings grown on a vertically positioned plate were used to measure root skewing angles. Angles of deviation of roots from the direction of the gravity vector were measured as described^6^.

### Immunostaining in Arabiodopsis roots

Cortical microtubule alignment was observed as described, with minor modifications to the protocol^37^. Anti-β-tubulin antibody (KMX-1, Merk Millipore: MAB3408) and Alexa Fluor 488-conjugated anti-mouse IgG (Thermo Fisher Scientific: A32723) were used as primary and secondary antibodies to label microtubules at dilutions of 1:1000 and 1:100, respectively. Fluorescent images were collected with a TCS SPE confocal laser scanning microscope (Leica Microsystems). Z-stack images were acquired and maximum intensity projections were produced according to the manufacturer’s instructions.

### Cell proliferation assay

Assays of HeLa cell proliferation were performed using a Cell counting kit-8 (Dojindo) according to the manufacturer’s instructions.

Growth of *Escherichia coli* DH5α was analyzed by measuring cell density in cultures with a spectrophotometer at a wavelength of 600 nm (OD600).

### Microscopic observation and image analyses of BY-2 cells

Cytoskeleton organization within transgenic BY-2 cells was observed using a fluorescence microscope equipped with a CSU-X1 confocal scanning unit (Yokogawa) and an sCMOS camera (Zyla, Andor Technology). Cytoskeleton density was evaluated by the image analyses that quantify the percentage of pixels for the cytoskeleton in the pixels for cytoplasm in the confocal images, using ImageJ software, as described^38,39^.

To detect cell death in BY-2 cells, an aliquot of the cell suspension was incubated with 0.05% Evans blue for 10 min at room temperature. The selective staining of dead cells with Evans blue depends on extrusion of the dye from living cells by an intact plasma membrane^40^. Stained cells were observed using brightfield microscopy (BX53, Olympus).

### Immunostaining in HeLa cells

HeLa cells were grown in DMEM supplemented with 10% FBS at 37 °C and 5% CO_2_ in a humidified incubator. Cells were treated with 100 µM KAND **11**, Kumamonamic acid **6**, Kumamonamide **1**, 100 ng/ml Colcemid (Gibco), or 100 ng/ml Nocodazole (Sigma) for 6 h at 37 °C. The cells were fixed with MetOH for 10 min and then with acetate for 5 min at room temperature. The fixed cells were incubated for 2 h with primary antibodies against β-tubulin (1D4A4, Proteintech: 66240-1) diluted in 0.5% BSA/PBS, washed three times with TBST, and then incubated for 1 h with Alexa Fluor 488 goat anti-mouse IgG (Thermo Fisher Scientific: A11001) and 15 ng/mL 4′, 6-diamidino-2-phenylindole (DAPI) diluted in 0.5% BSA/PBS. After washing three times with TBST, the stained cells were observed on a Nikon Eclipse Ti-E inverted microscope. Images were captured with a Hamamatsu ORCA-R2 cooled CCD camera using MetaMorph software (Molecular Devices).

## Supplementary Information

Supplementary Information 1.

Supplementary Information 2.
