# Supplementary material for: Discovery, characterization and functional improvement of kumamonamide as a novel plant growth inhibitor that disturbs plant microtubules
Source: Sci Rep. 2021 Mar 23;11:6077. doi: 10.1038/s41598-021-85501-1 (PMC7988157; doi:10.1038/s41598-021-85501-1)
Supplement: Supplementary file 1 — Supplementary Information 1. [file 41598_2021_85501_MOESM1_ESM.pdf]

Discovery, characterization and functional improvement of kumamonamide as a novel plant growth inhibitor that disturbs plant microtubules

Takashi Ishida, Haruna Yoshimura, Masatsugu Takekawa, Takumi Higaki, Takashi Ideue, Masaki Hatano, Masayuki Igarashi, Tokio Tani, Shinichiro Sawa, Hayato Ishikawa

General Remarks: All reactions were monitored by thin-layer chromatography using Merck 60 F254 precoated silica gel plates (0.25 mm thickness). Melting points were measured by Yanaco micromelting point apparatus. FTIR spectra were recorded on Shimadzu IR Affinity-1S. ^{1}H and ^{13}C NMR spectra were recorded on a JEOL ECX 500 FT-NMR spectrometer (500 MHz for ^{1}H NMR, 125 MHz for ^{13}C NMR) instrument. Data for ^{1}H NMR are reported as chemical shift (δ ppm), multiplicity (s = singlet, d = doublet, t = triplet, quint = quintet, dd = doubledoublet, dt = doubletriplet, m = multiplet), coupling constant (Hz), integration, and assignment. Data for ^{13}C NMR are reported as chemical shift. ESIMS and HRESIMS: recorded on a BRUKER impact Ⅱ.Flash chromatography was performed using silica gel 60N of Kanto Chemical Co. Int., Tokyo, Japan. Absorption spectra were measured by a Shimadzu UV-3600.

Isolation from *Streptomyces werraensis* culture supernatant

Streptomyces werraensis culture supernatant (60 mL) was extracted with 10% MeOH/EtOAc. The organic layer was evaporated under reduced pressure to give a residue (59.5 mg), and it was subjected to HPLC over reversed–phase column (SHISEIDO CAPCELL PAK C18 UG120, 5 µm, 10 mm I.D.×250 mm) with gradient elution (0–10 min: 90% H_2O/CH_3CN, 10–35 min: 90% H_2O/CH_3CN to 70% H_2O/CH_3CN (gradient), 35–45 min: 90% H_2O/EtOH, 45–155 min: 90% H_2O/EtOH to 100% EtOH (gradient), 155–200 min: 100% EtOH) at a flow rate of 1.5 mL/min to afford kumamonamide (**1**, 36.0 mg) as white amorphous powder.

Kumamonamide (**1**); ^{1}H-NMR (500 MHz, $CDCl_3$) δ 6.93 (t, *J* = 2.5 Hz, 1H), 6.76 (dd, *J* = 4.3, 1.8 Hz 1H), 6.05 (t, *J* = 3.8 Hz, 1H), 4.08 (s, 3H); ^{13}C-NMR (125 MHz, $CDCl_3$) δ 161.1, 121.0, 119.9, 112.2, 105.0, 68.3; ESI-HRMS $[M+H]^+$: calcd. for $[C_6H_9N_2O_2]^+$: 141.0659, found: 141.0663; IR ν_{max} 3451, 3414, 3173, 2938, 1603, 1593, 1537 cm^{-1}.

Total synthesis of kumamonamide

2-Aminopyridine 1-oxide (**3**)

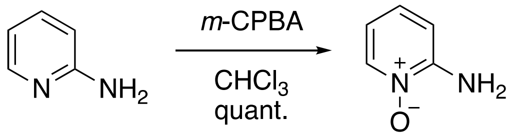

2 **3**

To a solution of 2-aminopyridine (**2**, 2.0 g, 22 mmol) in $CHCl_3$ (220 mL), *m*-chloroperbenzoic acid (*m*-CPBA, 5.6 g, 24 mmol) was added at 0 °C under Ar atmosphere. The reaction mixture was stirred for 9 h at room temperature. The resulting mixture was concentrated under reduced pressure. The residue was purified by SiO_2 column chromatography (25% MeOH/EtOAc) to afford 2-aminopyridine

1-oxide (**3**, 2.5 g, quantitative yield) as orange amorphous powder.

^{1}H-NMR (500 MHz, $CDCl_3$) δ 8.10 (d, *J* = 6.5 Hz, 1H), 7.13 (t, *J* = 8.0 Hz, 1H), 6.76 (d, *J* = 4.3 Hz, 1H), 6.64 (t, *J* = 3.5 Hz, 1H), 5.62 (br, 2H); ^{13}C-NMR (125 MHz, $CDCl_3$) δ 150.3, 137.9, 128.4, 113.7, 109.8; ESI-MS *m/z* 111 $[M+H]^+$.

2-Azidopyridine 1-oxide (**4**)

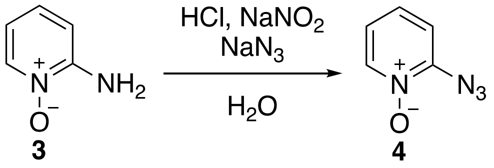

2.5 M Aqueous $NaNO_2$ (800 μL) was added dropwise to a solution of 2-aminopyridine 1-oxide (**3**, 200 mg, 1.8 mmol) in aqueous 10% HCl solution (60 mL) at 0 °C. The reaction mixture was stirred for 30 min, then a 2.5 M NaN_3 solution (800 μL) was added dropwise at 0 °C. The reaction mixture was stirred for 2 h at 0 °C. The resulting mixture was extracted with $CHCl_3$ (8×10 mL). The combined organic layers were dried over $MgSO_4$, filtered and concentrated under reduced pressure to provide the crude product of **4**. The crude materials of **4** was used in the next step without further purification.

1-Hydroxy-1*H*-pyrrole-2-carbonitrile (**5**)

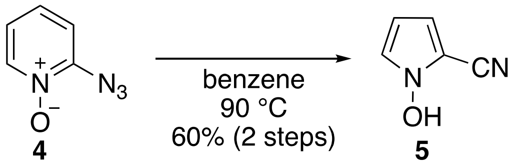

A solution of the crude materilas of **4** in benzene (4.5 mL) was heated in a sealed tube at 90 °C for 13 h under Ar atmosphere. The resulting mixture was concentrated under reduced pressure. The residue was purified by SiO_2 column chromatography (10% EtOAc/*n*-hexanes to 20% EtOAc/*n*-hexanes gradient) to afford 1-hydroxy-1*H*-pyrrole-2-carbonitrile (**5**, 128 mg, 65% yield) as light brown oil.

^{1}H-NMR (500 MHz, $CDCl_3$) δ 6.97 (dd, *J* = 2.0, 2.8 Hz, 1H), 6.62 (dd, *J* = 2.0, 4.8 Hz, 1H), 6.02 (dd, *J* = 2.8, 5.3 Hz, 1H); ^{13}C-NMR (125 MHz, $CDCl_3$) δ 123.4, 116.3, 112.7, 105.5, 100.4; ESI–MS *m/z* 107 [M–H].

1-Methoxy-1*H*-pyrrole-2-carbonitrile (**S1**)

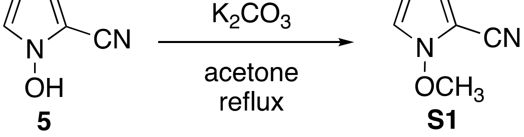

To a solution of 1-hydroxy-1*H*-pyrrole-2-carbonitrile (**5**, 98.0 mg, 0.91 mmol), and K_2CO_3 (140 mg, 1.0 mmol) in acetone (3.9 mL), methyl iodide (110 μL, 1.8 mmol) was added at room temperature. The reaction mixture was refluxed for 10.5 h under Ar atmosphere. After removal of excess amount of methyl iodide and acetone under reduced pressure, the solid residue was filtered with Et_2O and H_2O. The filtrate was extracted with Et_2O (3×10 mL). The combined organic layers were dried over $MgSO_4$, filtered and concentrated under reduced pressure to provide the crude materials of **S1**. The crude materials of **S1** was used in the next step without further purification.

1-Methoxy-1*H*-pyrrole-2-carboxylic acid (kumamonamic acid, **6**)

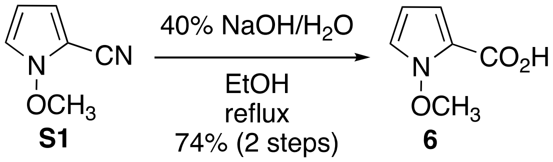

To a solution of the crude materials of S1 in EtOH (1.9 mL), 40% aqueous NaOH solution (1.2 mL) was added at room temperature. The reaction mixture was refluxed for 10 h. The resulting mixture was concentrated under reduced pressure. The residue was dissolved in water and pH of the resulting solution was adjusted to 3 by addition of 50% aqueous phosphoric acid. The aqueous layers were extracted with $CHCl_3$ (3×10 mL). The combined organic layers were dried over $MgSO_4$, filtered and concentrated under reduced pressure. The residue was purified by SiO_2 column chromatography (100% $CHCl_3$ to 5% MeOH/$CHCl_3$) to afford 1-methoxy-1*H*-pyrrole-2-carboxylic acid (kumamonamic acid, **6**, 104 mg, 74% yield) as light brown solid.

^{1}H-NMR (500 MHz, $CDCl_3$) δ 7.05 (t, J = 2.5 Hz, 1H), 6.92 (dd, J = 4.8, 2.3 Hz 1H), 6.06 (dd, J = 4.5, 2.5 Hz 1H), 4.11 (s, 3H); ^{13}C-NMR (125 MHz, $CDCl_3$) δ 161.2, 123.2, 117.1, 115.8, 104.8, 67.7; ESI-HRMS $[M+H]^+$: calcd. for $[C_6H_8NO_3]^+$: 142.0499, found: 142.0500; IR ν_{max} 3129, 2999, 2943, 1659, 1531, 1528 cm^{-1}; mp 80.5–82.1 °C.

Kumamonamide (**1**)

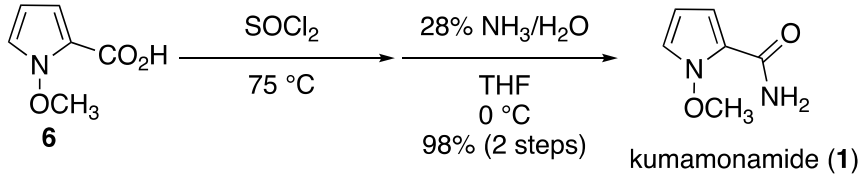

To a solution of 1-methoxy-1*H*-pyrrole-2-carboxylic acid (**6**, 50 mg, 0.35 mmol) and thionyl chloride (1.4 mL, 1.9 mmol) was added at room temperature under Ar atmosphere. The reaction mixture was stirred for 2.5 h at 75 °C under Ar. After cooling to room temperature, the resulting mixture was concentrated under reduced pressure and the residue was suspended in THF (2.0 mL). The suspension was poured into 28% aqueous NH_3 solution (3.9 mL) at 0 °C. The reaction mixture

was stirred for additional 2 h at 0 °C. The resulting mixture was concentrated under reduced pressure. The residue was suspended in $CHCl_3$ (10 mL) and 28% aqueous ammonia. The aqueous layers were extracted with $CHCl_3$ (3×10 mL). The combined organic layers were dried over $MgSO_4$, filtered and concentrated under reduced pressure. The residue was purified by SiO_2 column chromatography (3% MeOH/$CHCl_3$) to afford kumamonamide (**1**, 49 mg, 98% yield) as white amorphous powder. All spectral data of provided kumamonamide (**1**) were identical with naturally occurring **1**.

Preparation of kumamonamide derivatives

1-Hydroxy-1*H*-pyrrole-2-carboxylic acid (**7**)

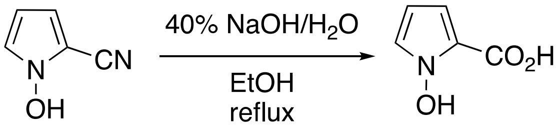

5 **7**

To a solution of 1-hydroxy-1*H*-pyrrole-2-carbonitrile (**5**, 100 mg, 0.925 mmol) in EtOH (1.97 mL), 40% NaOH/H_2O (1.27 mL) was added at room temperature. The reaction mixture was refluxed for 10 h. The resulting mixture was concentrated under reduced pressure. The residue was dissolved in water and pH of the resulting solution was adjusted to 3 by addition of 50% aqueous phosphoric acid. The aqueous layers were extracted with ether (3×10 mL). The combined organic layers were dried over $MgSO_4$, filtered and concentrated under reduced pressure. The residue was purified by SiO_2 column chromatography (50% EtOAc/*n*-hexanes) to afford 1-hydroxy-1*H*-pyrrole-2-carboxylic acid (**7**, 62.9 mg, 48% yield) as light brown amorphous powder.

^{1}H-NMR (500 MHz, $CDCl_3$) δ 7.07 (t, J = 2.3 Hz, 1H), 6.83–6.84 (m, 1H), 6.07 (dd, J = 5.0, 2.5 Hz 1H); ^{13}C-NMR (125 MHz, $CDCl_3$) δ 167.3, 120.9, 113.0, 104.5, 77.1; ESI-HRMS $[M+H]^+$: calcd. for $[C_5H_6NO_3]^+$: 128.0342, found: 128.0342; IR ν_{max} 2918, 2851, 1659 cm^{-1}.

1-Propoxy-1*H*-pyrrole-2-carboxylic acid (**8**)

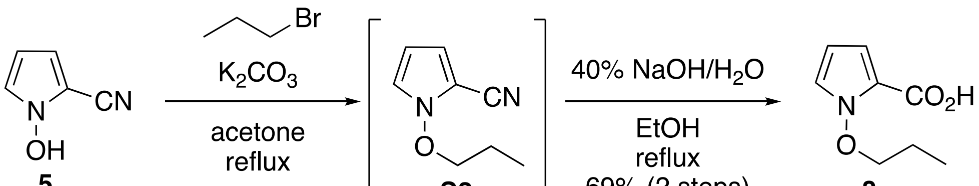

5 **S2** **8**

To a solution of mixture of 1-hydroxy-1*H*-pyrrole-2-carbonitrile (**5**, 50 mg, 0.46 mmol) and K_2CO_3 (70 mg, 0.51 mmol) in acetone (2.0 mL), 1-bromopropane (84 μL, 0.93 mmol) was added at room temperature under Ar atmosphere. The reaction mixture was refluxed for 5.5 h under Ar. After removal of excess amount of 1-bromopropane and acetone under reduced pressure, the solid residue was filtered with Et_2O and H_2O. The filtrate was extracted with Et_2O (3×10 mL). The combined organic

layers were dried over $MgSO_4$, filtered and concentrated under reduced pressure to provide the crude materials of **S2**. The crude materials of **S2** was used in the next step without further purification.

To a solution of the crude materials of **S2** in EtOH (980 μL), 40% aqueous NaOH (630 μL) was added at room temperature. The reaction mixture was refluxed for 9 h. After cooling to room temperature, the resulting mixture was concentrated under reduced pressure. The residue was dissolved in water and pH of the resulting solution was adjusted to 3 by addition of 50% aqueous phosphoric acid. The aqueous layers were extracted with $CHCl_3$ (3×10 mL). The combined organic layers were dried over $MgSO_4$, filtered and concentrated under reduced pressure. The residue was purified by SiO_2 column chromatography (1% MeOH/$CHCl_3$) to afford 1-propoxy-1*H*-pyrrole-2-carboxylic acid (**8**, 54 mg, 69% yield) as brown solid.

^{1}H-NMR (500 MHz, $CDCl_3$) δ 7.02–7.01 (m, 1H), 6.92 (dd, J = 4.5, 2.5 Hz, 1H), 6.05–6.03 (m, 1H), 4.22 (t, J = 6.8 Hz, 2H), 1.79 (sext, J = 7.1 Hz, 2H), 1.05 (dt, J = 7.5, 1 Hz, 3H); ^{13}C-NMR (125 MHz, $CDCl_3$) δ 164.6, 124.2, 117.6, 116.0, 104.8, 82.2, 21.4, 10.3; ESI-HRMS $[M+H]^+$: calcd. for $[C_8H_{12}NO_3]^+$: 170.0812, found: 170.0799 ; IR ν_{max} 3136, 2963, 2922, 2876, 1661, 1539, 1526 cm^{-1}; mp 49.1–52.0 °C.

1-(Pentyloxy)-1*H*-pyrrole-2-carboxylic acid (**9**)

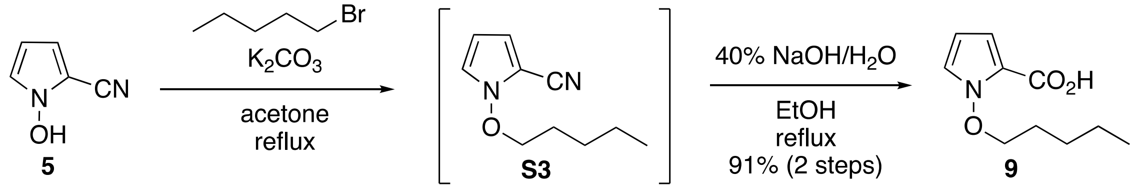

To a solution of mixture of 1-hydroxy-1*H*-pyrrole-2-carbonitrile (**5**, 49 mg, 0.45 mmol) and K_2CO_3 (70 mg, 0.51 mmol) in acetone (2.0 mL), 1-bromopentane (110 μL, 0.93 mmol) was added at room temperature under Ar atmosphere. The reaction mixture was refluxed for 5.5 h under Ar. After removal of excess amount of 1-bromopentane and acetone under reduced pressure, the solid residue was filtered with Et_2O and H_2O. The filtrate was extracted with Et_2O (3×10 mL). The combined organic layers were dried over $MgSO_4$, filtered and concentrated under reduced pressure to provide the crude materials of **S3**. The crude materials of **S3** was used in the next step without further purification.

To a solution of the crude materials of **S3** in EtOH (960 μL), 40% aqueous NaOH (620 μL) was added at room temperature. The reaction mixture was refluxed for 9 h. After cooling to room temperature, the resulting mixture was concentrated under reduced pressure. The residue was dissolved in water and pH of the resulting solution was adjusted to 3 by addition of 50% aqueous phosphoric acid. The aqueous layers were extracted with $CHCl_3$ (3×10 mL). The combined organic layers were dried over $MgSO_4$, filtered and concentrated under reduced pressure. The residue was purified by SiO_2 column chromatography (1% MeOH/$CHCl_3$) to afford 1-(pentyloxy)-1*H*-pyrrole-2-

carboxylic acid (**9**, 81 mg, 91% yield) as yellow oil.

^{1}H-NMR (500 MHz, $CDCl_3$) δ 7.02 (t, *J* = 2.8 Hz, 1H), 6.92 (dd, *J* = 4.5, 2.5 Hz, 1H), 6.04 (dd, *J* = 4.8, 2.8 Hz, 1H), 4.26 (t, *J* = 7.0 Hz, 2 H), 1.77 (quint, *J* = 7.0 Hz, 2H), 1.48–1.36 (m, 4H), 0.94 (t, *J* = 7.0 Hz, 3H); ^{13}C-NMR (125 MHz, $CDCl_3$) δ 164.6, 124.2, 117.6, 116.0, 104.8, 80.9, 27.9, 27.7, 22.6, 14.1; ESI-HRMS $[M+H]^+$: calcd. for $[C_{10}H_{16}NO_3]^+$: 198.1125, found: 198.1100; IR ν_{max} 3123, 2957, 2930, 2859, 1682, 1661, 1541, 1530 cm^{-1}.

1-(Heptloxy)-1*H*-pyrrole-2-carboxylic acid (**10**)

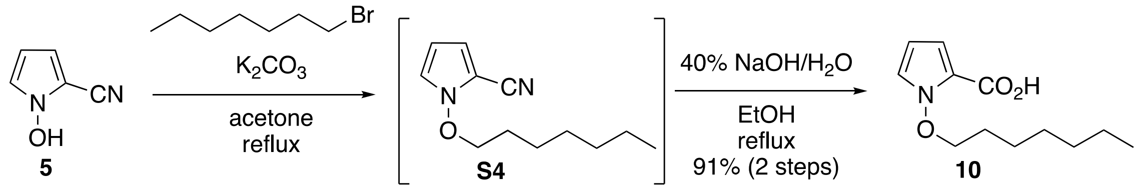

To a solution of mixture of 1-hydroxy-1*H*-pyrrole-2-carbonitrile (**5**, 50 mg, 0.46 mmol) and K_2CO_3 (70 mg, 0.51 mmol) in acetone (2.0 mL), 1-bromoheptane (150 μL, 0.93 mmol) was added at room temperature under Ar atmosphere. The reaction mixture was refluxed for 1.5 h under Ar. After removal of excess amount of 1-bromoheptane and acetone under reduced pressure, the solid residue was filtered with Et_2O and H_2O. The filtrate was extracted with Et_2O (3×10 mL). The combined organic layers were dried over $MgSO_4$, filtered and concentrated under reduced pressure to provide the crude materials of **S4**. The crude materials of **S4** was used in the next step without further purification.

To a solution of the crude materials of **S4** in EtOH (980 μL), 40% aqueous NaOH (630 μL) was added at room temperature. The reaction mixture was refluxed for 17 h. After cooling to room temperature, the resulting mixture was concentrated under reduced pressure. The residue was dissolved in water and pH of the resulting solution was adjusted to 3 by addition of 50% aqueous phosphoric acid. The aqueous layers were extracted with $CHCl_3$ (3×10 mL). The combined organic layers were dried over $MgSO_4$, filtered and concentrated under reduced pressure. The residue was purified by SiO_2 column chromatography (1% MeOH/$CHCl_3$) to afford 1-(heptyloxy)-1*H*-pyrrole-2-carboxylic acid (**10**, 65 mg, 91% yield) as yellow oil.

^{1}H-NMR (500 MHz, $CDCl_3$) δ 7.02 (t, *J* = 2.8 Hz, 1H), 6.92 (dd, *J* = 4.5, 2.0 Hz, 1H), 6.04 (dd, *J* =4.8, 2.8 Hz, 1H), 4.30 (t, *J* = 6.8 Hz, 2H), 1.76 (quint, *J* = 7.1 Hz, 2H), 1.46 (quint, *J* = 7.4 Hz, 2H), 1.38–1.26 (m, 6H), 0.90 (t, *J* = 6.8 Hz, 3H); ^{13}C-NMR (125 MHz, $CDCl_3$) δ 164.3, 123.9, 117.4, 115.7, 104.5, 80.7, 31.6, 28.9, 27.8, 25.5, 22.4, 14.0; ESI-HRMS $[M+H]^+$: calcd. for $[C_{12}H_{20}NO_3]^+$: 226.1438, found: 226.1421; IR ν_{max} 3132, 2955, 2920, 2855, 1666, 1539, 1526 cm^{-1}.

1-(Nonyloxy)-1*H*-pyrrole-2-carboxylic acid (**11**)

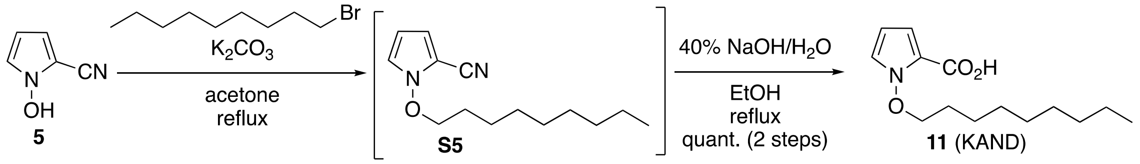

To a solution of mixture of 1-hydroxy-1*H*-pyrrole-2-carbonitrile (**5**, 50 mg, 0.46 mmol) and K_2CO_3 (70 mg, 0.51 mmol) in acetone (2.0 mL), 1-bromononane (180 μL, 0.93 mmol) was added at room temperature under Ar atmosphere. The reaction mixture was refluxed for 1.5 h under Ar. After removal of acetone under reduced pressure, the solid residue was filtered with Et_2O and H_2O. The filtrate was extracted with Et_2O (3×10 mL). The combined organic layers were dried over $MgSO_4$, filtered and concentrated under reduced pressure to provide the crude materials of **S5**. The crude materials of **S5** was used in the next step without further purification.

To a solution of the crude materials of **S5** in EtOH (980 μL), 40% aqueous NaOH (630 μL) was added at room temperature. The reaction mixture was refluxed for 19 h. After cooling to room temperature, the resulting mixture was concentrated under reduced pressure. The residue was dissolved in water and pH of the resulting solution was adjusted to 3 by addition of 50% aqueous phosphoric acid. The aqueous layers were extracted with $CHCl_3$ (3×10 mL). The combined organic layers were dried over $MgSO_4$, filtered and concentrated under reduced pressure. The residue was purified by SiO_2 column chromatography (1% MeOH/$CHCl_3$) to afford 1-(nonyloxy)-1*H*-pyrrole-2-carboxylic acid (**11**, 121 mg, quant.) as yellow oil.

^{1}H-NMR (500 MHz, $CDCl_3$) δ 7.01 (t, *J* = 2.8 Hz, 1H), 6.92 (dd, *J* = 4.5, 2.5 Hz, 1H), 6.04 (dd, *J* = 4.75, 2.8 Hz, 1H), 4.26 (t, *J* = 7.0 Hz, 2H), 1.76 (quint, *J* = 7.1 Hz, 2H), 1.47-1.43 (m, 2H), 1.36–1.23 (m, 10H), 0.89 (t, *J* = 7.0 Hz, 3H); ^{13}C-NMR (125 MHz, $CDCl_3$) δ 164.5, 124.1, 117.6, 115.9, 104.7, 80.9, 32.0, 29.6, 29.5, 29.4, 28.0, 25.8, 22.8, 14.2; ESI-HRMS $[M+H]^+$: calcd. for $[C_{14}H_{24}NO_3]^+$: 254.1751, found: 254.1747; IR ν_{max} 2955, 2924, 2853, 1682, 1659, 1539, 1528 cm^{-1}.

<u>1-(Undecyloxy)-1*H*-pyrrole-2-carbonitrile (**S6**)</u>

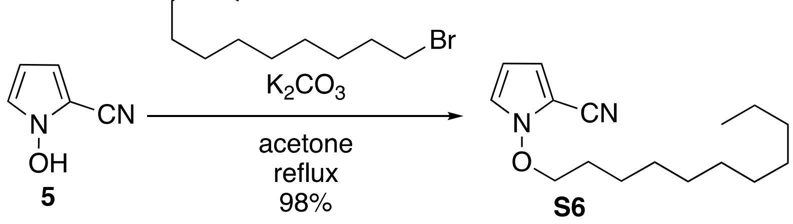

To a solution of 1-hydroxy-1*H*-pyrrole-2-carbonitrile (**5**, 52 mg, 0.48 mmol) and K_2CO_3 (73 mg, 0.53 mmol) in acetone (2.1 mL), 1-bromoundecane (220 μL, 0.96 mmol) was added at room temperature under Ar atmosphere. The reaction mixture was refluxed for 2 h under argon. After removal of acetone under reduced pressure, the solid residue was filtered with Et_2O and H_2O. The

filtrate was extracted with Et_2O (3×10 mL). The combined organic layers were dried over $MgSO_4$, filtered and concentrated under reduced pressure.The residue was purified by SiO_2 column chromatography (5% EtOAc/*n*-hexanes) to afford 1-(undecyloxy)-1*H*-pyrrole-2- carbonitrile (**S6**, 116 mg, 98%) as colorless oil.

^{1}H-NMR (500 MHz, $CDCl_3$) δ 6.94 (t, *J* = 2.8 Hz, 1H), 6.59 (dd, *J* = 4.5, 3.0 Hz, 1H), 6.03 (dd, *J* = 4.5, 3.0 Hz, 1H), 4.24 (t, *J* = 6.5 Hz, 2H), 1.75 (quint, *J* = 7.1 Hz, 2H), 1.45 (quint, *J* = 7.4 Hz, 2H), 1.33–1.26 (m, 14H), 0.87 (t, *J* = 7.0 Hz, 3H); ^{13}C-NMR (125 MHz, $CDCl_3$) δ 121.5, 115.8, 112.2, 105.8, 100.7, 81.9, 32.2, 29.9, 29.9, 29.8, 29.6, 29.6, 28.2, 25.9, 23.0, 14.4; ESI-HRMS $[M+H]^+$: calcd. for $[C_{16}H_{27}N_2O]^+$: 263.2118, found: 263.2094; IR ν_{max} 3129, 2953, 2924, 2853, 2224, 1526 cm^{-1}.

1-(Undecyloxy)-1*H*-pyrrole-2-carboxylic acid (**12**)

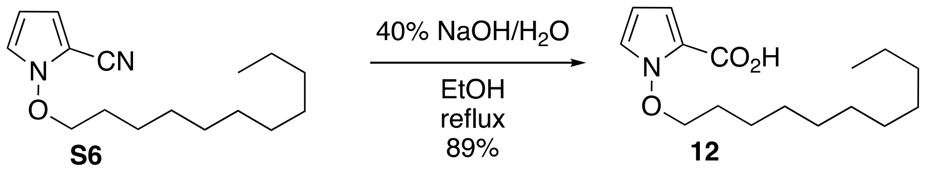

To a solution of 1-(undecyloxy)-1*H*-pyrrole-2-carbonitrile (**S6**, 80 mg, 0.32 mmol) in EtOH (690 μL), 40% aqueous NaOH (440 μL) was added at room temperature. The reaction mixture was refluxed for 17 h. After cooling to room temperature, the resulting mixture was concentrated under reduced pressure. The residue was dissolved in water and pH of the resulting solution was adjusted to 3 by addition of 50% aqueous phosphoric acid. The aqueous layers were extracted with $CHCl_3$ (3×10 mL). The combined organic layers were dried over $MgSO_4$, filtered and concentrated under reduced pressure. The residue was purified by SiO_2 column chromatography (3% MeOH/$CHCl_3$) to afford 1-(undecyloxy)-1*H*-pyrrole-2-carboxylic acid (**12**, 81.0 mg, 89%) as yellow solid.

^{1}H-NMR (500 MHz, $CDCl_3$) δ 7.01 (d, *J* = 1.5 Hz, 1H), 6.92 (t, *J* = 2.5 Hz, 1H), 6.04 (s, 1H), 4.26 (t, *J* = 5.5 Hz, 2H), 1.77–1.75 (m, 2H), 1.46-1.45 (m, 2H), 1.28 (s, 14H), 0.89 (t, *J* = 6.0 Hz, 3H); ^{13}C-NMR (125 MHz, $CDCl_3$) δ 164.7, 124.1, 117.6, 116.0, 104.7, 80.8, 32.0, 29.8, 29.7, 29.7, 29.5, 29.5, 28.0, 25.8, 22.8, 14.2; ESI-HRMS $[M+H]^+$: calcd. for $[C_{16}H_{28}NO_3]^+$: 282.2064, found: 282.2091; IR ν_{max} 3130, 2953, 2918, 2849, 1686, 1661, 1530 cm^{-1}; mp 39.9–41.8 °C.

1-(Tridecyloxy)-1*H*-pyrrole-2-carbonitrile (**S7**)

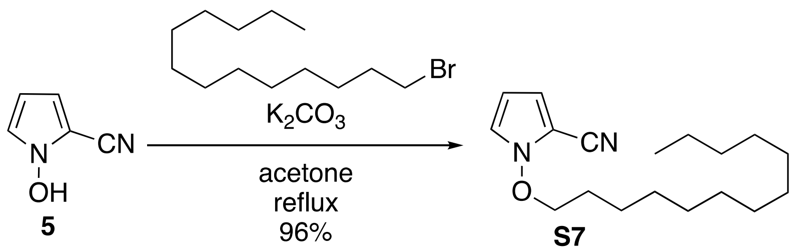

To a solution of 1-hydroxy-1*H*-pyrrole-2-carbonitrile (**5**, 50 mg, 0.46 mmol) and K_2CO_3 (70 mg, 0.51 mmol) in acetone (2.0 mL), 1-bromotridecane (240 μL, 0.93 mmol) was added at room temperature under Ar atmosphere. The reaction mixture was refluxed for 1 h under argon. After removal of acetone under reduced pressure, the solid residue was filtered with Et_2O and H_2O. The filtrate was extracted with Et_2O (3×10 mL). The combined organic layers were dried over $MgSO_4$, filtered and concentrated under reduced pressure. The residue was purified by SiO_2 column chromatography (5% EtOAc/*n*-hexanes) to afford 1-(tridecyloxy)-1*H*-pyrrole-2- carbonitrile (**S7**, 128 mg, 96%) as colorless oil.

^{1}H-NMR (500 MHz, $CDCl_3$) δ 6.93 (dd, *J* = 3.0, 2.0 Hz, 1H), 6.57 (dd, *J* = 4.8, 2.3 Hz, 1H), 6.01 (dd, *J* = 4.5, 3.5 Hz, 1H), 4.23 (t, *J* = 6.5 Hz, 2H), 1.73 (quint, *J* = 7.1 Hz, 2H), 1.44 (quint, *J* = 7.4 Hz, 2H), 1.25 (s, 18H), 0.86 (t, *J* = 7.0 Hz, 3H); ^{13}C-NMR (125 MHz, $CDCl_3$) δ 121.0, 115.2, 111.6, 105.2, 100.1, 81.3, 31.7, 29.4, 29.4, 29.4, 29.3, 29.2, 29.1, 29.0, 27.6, 25.3, 22.4, 14.0; ESI-HRMS $[M+H]^+$: calcd. for $[C_{18}H_{31}N_2O]^+$: 291.2431, found: 291.2399; IR ν_{max} 3129, 2922, 2853, 2224, 1526 cm^{-1}.

1-(Tridecyloxy)-1*H*-pyrrole-2-carboxylic acid (**13**)

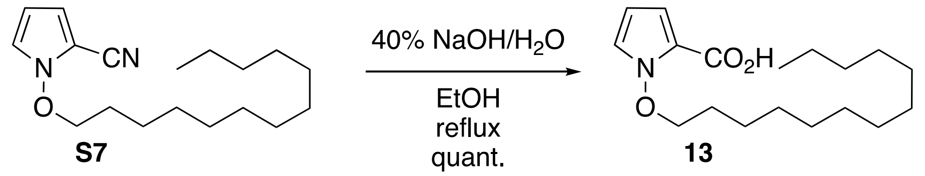

To a solution of 1-(undecyloxy)-1*H*-pyrrole-2-carbonitrile (**S7**, 80 mg, 0.28 mmol) in EtOH (590 μL), 40% aqueous NaOH (370 μL) was added at room temperature. The reaction mixture was refluxed for 16 h. After cooling to room temperature, the resulting mixture was concentrated under reduced pressure. The residue was dissolved in water and pH of the resulting solution was adjusted to 3 by addition of 50% aqueous phosphoric acid. The aqueous layers were extracted with $CHCl_3$ (3×10 mL). The combined organic layers were dried over $MgSO_4$, filtered and concentrated under reduced pressure. The residue was purified by SiO_2 column chromatography (3% MeOH/$CHCl_3$) to afford 1-(tridecyloxy)-1*H*-pyrrole-2-carboxylic acid (**13**, 85 mg, quant.) as yellow amorphous powder.

^{1}H-NMR (500 MHz, $CDCl_3$) δ 7.01 (t, *J* = 2.3 Hz, 1H), 6.92 (dd, *J* = 4.3, 1.8 Hz, 1H), 6.04 (dd, *J* = 4.0, 2.5 Hz, 1H), 4.26 (t, *J* = 6.5 Hz, 2H), 1.76 (quint, *J* = 7.1 Hz, 2H), 1.46 (quint, *J* = 7.3 Hz, 2H), 1.27 (s, 18H), 0.89 (t, *J* = 6.8 Hz, 3H); ^{13}C-NMR (125 MHz, $CDCl_3$) δ 164.4, 124.0, 117.4, 115.7, 104.5, 80.6, 31.8, 29.6, 29.5, 29.5, 29.4, 29.3, 29.3, 27.8, 25.6, 22.6, 14.0; ESI-HRMS $[M+H]^+$: calcd. for $[C_{18}H_{32}NO_3]^+$: 310.2377, found: 310.2376; IR ν_{max} 2916, 2849, 1687, 1663, 1541, 1530 cm^{-1}.

1-(Pentadecyloxy)-1*H*-pyrrole-2-carbonitrile (**S8**)

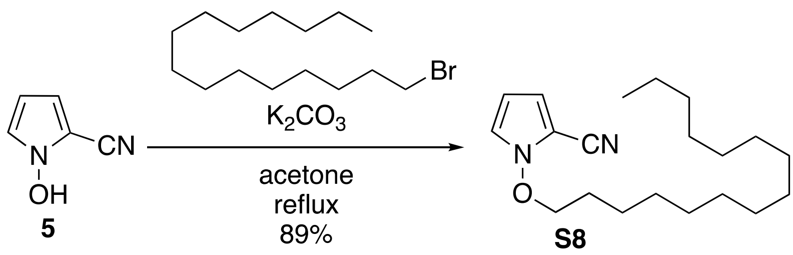

To a solution of 1-hydroxy-1*H*-pyrrole-2-carbonitrile (**5**, 50 mg, 0.46 mmol) and K_2CO_3 (70 mg, 0.51 mmol) in acetone (2.0 mL), 1-bromopentadecane (270 μL, 0.93 mmol) was added at room temperature under Ar atmosphere. The reaction mixture was refluxed for 1 h under argon. After removal of acetone under reduced pressure, the solid residue was filtered with Et_2O and H_2O. The filtrate was extracted with Et_2O (3×10 mL). The combined organic layers were dried over $MgSO_4$, filtered and concentrated under reduced pressure. The residue was purified by SiO_2 column chromatography (5% EtOAc/*n*-hexanes) to afford 1-(pentadecyloxy)-1*H*-pyrrole-2- carbonitrile (**S8**, 132 mg, 89%) as colorless oil.

^{1}H-NMR (500 MHz, $CDCl_3$) δ 6.93 (dd, *J* = 2.8, 1.8 Hz, 1H), 6.59–6.57 (m, 1H), 6.02 (dd, *J* = 4.5, 3.5 Hz, 1H), 4.24 (t, *J* = 6.5 Hz, 2H), 1.74 (quint, *J* = 7.3 Hz, 2H), 1.45 (quint, *J* = 7.3 Hz, 2H), 1.33–1.25 (m, 22H), 0.87 (t, *J* = 7.0 Hz, 3H); ^{13}C-NMR (125 MHz, $CDCl_3$) δ 121.5, 115.9, 112.3, 105.8, 100.7, 82.0, 32.3, 30.1, 30.0, 30.0, 29.9, 29.8, 29.7, 29.7, 28.3, 26.0, 23.1, 14.5; ESI-HRMS $[M+H]^+$: calcd. for $[C_{20}H_{35}N_2O]^+$: 319.2744, found: 319.2709; IR ν_{max} 3127, 2922, 2853, 2224, 1526 cm^{-1}.

<u>1-(Pentadecyloxy)-1*H*-pyrrole-2-carboxylic acid (**14**)</u>

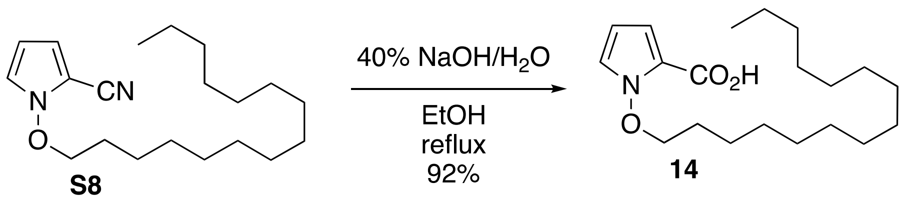

To a solution of 1-(pentadecyloxy)-1*H*-pyrrole-2-carbonitrile (**S8**, 20 mg, 63 μmol) in EtOH (130 μL), 40% aqueous NaOH (86 μL) was added at room temperature. The reaction mixture was refluxed for 18.5 h. After cooling to room temperature, the resulting mixture was concentrated under reduced pressure. The residue was dissolved in water and pH of the resulting solution was adjusted to 3 by addition of 50% aqueous phosphoric acid. The aqueous layers were extracted with $CHCl_3$ (3×10 mL). The combined organic layers were dried over $MgSO_4$, filtered and concentrated under reduced pressure. The residue was purified by SiO_2 column chromatography (3% MeOH/$CHCl_3$) to afford 1-(pentadecyloxy)-1*H*-pyrrole-2-carboxylic acid (**14**, 20 mg, 92%) as light yellow solid.

^{1}H-NMR (500 MHz, $CDCl_3$) δ 7.01 (t, *J* = 2.5 Hz, 1H), 6.91 (dd, *J* = 4.5, 2.5 Hz, 1H), 6.04 (dd, *J* = 4.5, 3.0 Hz, 1H), 4.25 (t, *J* = 6.5 Hz, 2H), 1.76 (quint, *J* = 7.1 Hz, 2H), 1.45 (quint, *J* = 7.3 Hz, 2H),

1.31–1.26 (m, 22H), 0.88 (t, *J* = 7.0 Hz, 3H); ^{13}C-NMR (125 MHz, $CDCl_3$) δ 164.6, 124.4, 117.9, 116.2, 105.0, 81.2, 32.3, 30.1, 30.1, 30.0, 29.9, 29.8, 28.3, 26.1, 23.1, 14.5; ESI-HRMS [M+H]$^+$: calcd. for $[C_{20}H_{36}NO_3]^+$: 338.2690, found: 338.2670; IR ν_{max} 2953, 2916, 2849, 1682, 1663, 1539, 1530 cm^{-1}; mp 59.5–61.5 °C.

<u>1-(Benzyloxy)-1*H*-pyrrole-2-carboxylic acid (**15**)</u>

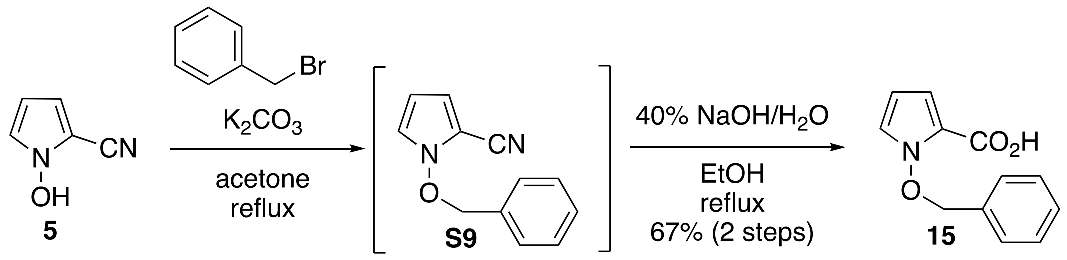

To a solution of mixture of 1-hydroxy-1*H*-pyrrole-2-carbonitrile (**5**, 50 mg, 0.46 mmol) and K_2CO_3 (71 mg, 0.51 mmol) in acetone (2.0 mL), benzyl bromide (110 μL, 0.93 mmol) was added at room temperature under Ar atmosphere. The reaction mixture was refluxed for 30 min under Ar. After removal of acetone under reduced pressure, the solid residue was filtered with Et_2O and H_2O. The filtrate was extracted with Et_2O (3×10 mL). The combined organic layers were dried over $MgSO_4$, filtered and concentrated under reduced pressure to provide the crude materials of **S9**. The crude materials of **S9** was used in the next step without further purification.

To a solution of the crude materials of **S9** in EtOH (821 μL), 40% aqueous NaOH (529 μL) was added at room temperature. The reaction mixture was refluxed for 30 min. After cooling to room temperature, the resulting mixture was concentrated under reduced pressure. The residue was dissolved in water and pH of the resulting solution was adjusted to 3 by addition of 50% aqueous phosphoric acid. The aqueous layers were extracted with $CHCl_3$ (3×10 mL). The combined organic layers were dried over $MgSO_4$, filtered and concentrated under reduced pressure. The residue was purified by SiO_2 column chromatography (1% MeOH/$CHCl_3$) to afford 1-(benzyloxy)-1*H*-pyrrole-2-carboxylic acid (**15**, 56.4 mg, 67% yield) as brown amorphous powder.

^{1}H-NMR (500 MHz, $CDCl_3$) δ 7.45–7.39 (m, 5H), 6.98 (dd, *J* = 4.5, 2.5 Hz, 1H), 6.76 (t, *J* = 2.5 Hz, 1H), 5.98 (dd, *J* = 4.3, 2.3 Hz, 1H), 5.27 (s, 2H); ^{13}C-NMR (125 MHz, $CDCl_3$) δ 164.4, 134.1, 130.3, 130.1, 129.4, 128.8, 128.6, 124.9, 117.5, 116.3, 104.6, 82.3; ESI-HRMS [M+H]$^+$: calcd. for $[C_{12}H_{12}NO_3]^+$: 218.0812, found: 218.0782; IR ν_{max} 3134, 1659, 1541, 1530 cm^{-1}.

<u>1-(Naphthalen-2-ylmethoxy)-1*H*-pyrrole-2-carbonitrile (**S10**)</u>

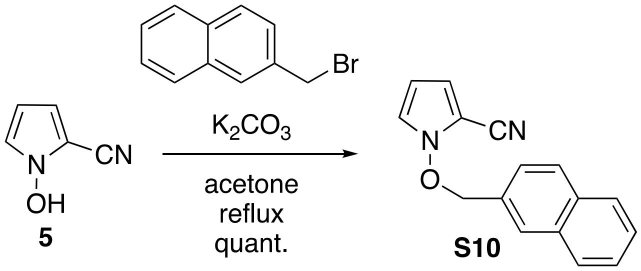

To a solution of 1-hydroxy-1*H*-pyrrole-2-carbonitrile (**5**, 50 mg, 0.46 mmol) and K_2CO_3 (70 mg, 0.51 mmol) in acetone (2.0 mL), 1-bromopentadecane (270 μL, 0.93 mmol) was added at room temperature under Ar atmosphere. The reaction mixture was refluxed for 1 h under argon. After removal of acetone under reduced pressure, the solid residue was filtered with Et_2O and H_2O. The filtrate was extracted with Et_2O (3×10 mL). The combined organic layers were dried over $MgSO_4$, filtered and concentrated under reduced pressure. The residue was purified by SiO_2 column chromatography (5% EtOAc/*n*-hexanes to 10% EtOAc/*n*-hexanes) to afford 1- (naphthalen-2-ylmethoxy)-1*H*-pyrrole-2-carbonitrile (**S10**, 115 mg, quant.) as white solid.

^{1}H-NMR (500 MHz, $CDCl_3$) δ 7.89 (t, *J* = 9.5 Hz, 2H), 7.84 (d, *J* = 8.0 Hz, 1H), 7.77 (s, 1H), 7.56–7.52 (m, 3H), 6.69 (d, *J* = 1.5 Hz, 1H), 6.61 (dd, *J* = 4.8, 1.8 Hz, 1H), 5.93 (dd, *J* = 4.8, 2.8 Hz, 1H), 5.36 (s, 2H); ^{13}C-NMR (125 MHz, $CDCl_3$) δ 133.7, 133.0, 130.4, 129.7, 128.7, 128.2, 127.8, 127.0, 126.7, 126.5, 121.9, 115.7, 112.1, 105.3, 100.2, 82.9; ESI-HRMS $[M+H]^+$: calcd. for $[C_{16}H_{13}N_2O]^+$: 249.1022, found: 249.0986; IR ν_{max} 2218, 1535, 1516, 1508 cm-1; mp 65.4–67.8 °C.

1-(Naphthalen-2-ylmethoxy)-1*H*-pyrrole-2-carboxylic acid (**16**)

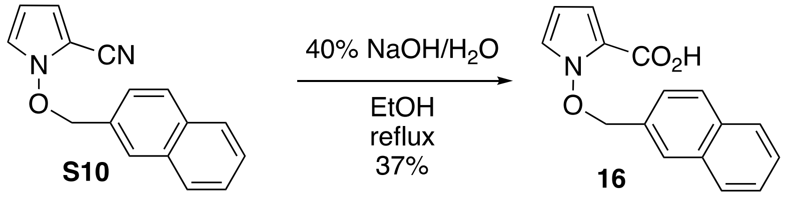

To a solution of 1-(naphthalen-2-ylmethoxy)-1*H*-pyrrole-2-carbonitrile (**S10**, 20 mg, 81 μmol) in EtOH (170 μL), 40% aqueous NaOH (110 μL) was added at room temperature. The reaction mixture was refluxed for 112 h. After cooling to room temperature, the resulting mixture was concentrated under reduced pressure. The residue was dissolved in water and pH of the resulting solution was adjusted to 3 by addition of 50% aqueous phosphoric acid. The aqueous layers were extracted with $CHCl_3$ (3×10 mL). The combined organic layers were dried over $MgSO_4$, filtered and concentrated under reduced pressure. The residue was purified by SiO_2 column chromatography (5% MeOH/$CHCl_3$) to afford 1-(naphthalen-2-ylmethoxy)-1*H*-pyrrole-2-carboxylic acid (**16**, 7.9 mg, 37%) as brown amorphous powder.

^{1}H-NMR (500 MHz, $CDCl_3$) δ 7.88–7.82 (m, 4H), 7.59 (d, *J* = 8.0 Hz, 1H), 7.51 (quint, *J* = 6.5 Hz,

2H), 6.93 (dd, J = 4.3, 1.3 Hz, 1H), 6.72 (t, J = 2.5 Hz, 1H), 5.93 (t, J = 3.8 Hz, 1H), 5.41 (s, 2H); ^{13}C-NMR (125 MHz, $CDCl_3$) δ 163.4, 133.7, 133.2, 131.6, 129.7, 128.7, 128.3, 127.9, 127.1, 126.8, 126.5, 124.9, 116.1, 104.6, 94.2, 82.4; ESI-HRMS $[M+H]^+$: calcd. for $[C_{16}H_{14}NO_3]^+$: 268.0968, found: 268.0968; IR ν_{max} 3132, 2922, 2853, 1682, 1659, 1541, 1528 cm^{-1}.

1-((4-Phenoxybenzyl)oxy)-1*H*-pyrrole-2-carbonitrile (**S11**)

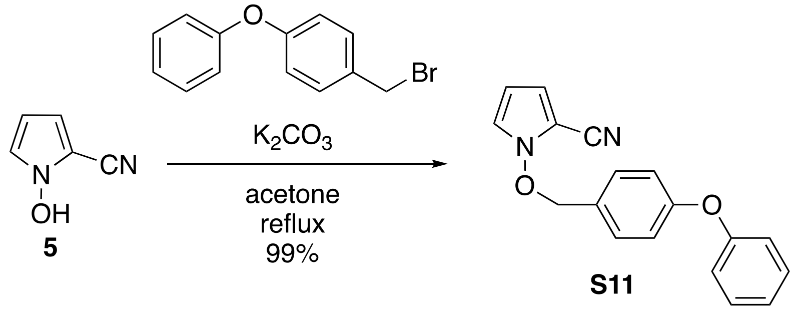

To a solution of 1-hydroxy-1*H*-pyrrole-2-carbonitrile (**5**, 100 mg, 0.93 mmol) and K_2CO_3 (140 mg, 1.0 mmol) in acetone (4.0 mL), 4-phenoxybenzyl bromide (240 mg, 0.93 mmol) was added at room temperature under Ar atmosphere. The reaction mixture was refluxed for 1.5 h under argon. After removal of acetone under reduced pressure, the solid residue was filtered with Et_2O and H_2O. The filtrate was extracted with Et_2O (3×10 mL). The combined organic layers were dried over $MgSO_4$, filtered and concentrated under reduced pressure. The residue was purified by SiO_2 column chromatography (5% EtOAc/*n*-hexanes to 10% EtOAc/*n*-hexanes) to afford 1-((4-phenoxybenzyl)oxy)-1*H*-pyrrole-2-carbonitrile (**S11**, 266 mg, 99%) as colorless oil.

^{1}H-NMR (500 MHz, $CDCl_3$) δ 7.38–7.31 (m, 4H), 7.15 (t, J = 7.5 Hz, 1H), 7.05–6.99 (m, 4H), 6.74 (t, J = 2.5 Hz, 1H), 6.61 (dd, J = 4.8, 1.8 Hz, 1H), 5.99 (dd, J = 4.5, 2.5 Hz, 1H), 5.17 (s, 2H); ^{13}C-NMR (125 MHz, $CDCl_3$) δ 159.0, 156.5, 131.9, 130.0, 127.5, 123.9, 121.8, 119.5, 118.6, 115.7, 112.0, 105.5, 100.4, 82.4; ESI-HRMS $[M+H]^+$: calcd. for $[C_{18}H_{15}N_2O_2]^+$: 291.1128, found: 291.1096; IR ν_{max} 3127, 2222, 1612, 1587, 1508 cm^{-1}.

1-((4-Phenoxybenzyl)oxy)-1*H*-pyrrole-2-carboxylic acid (**17**)

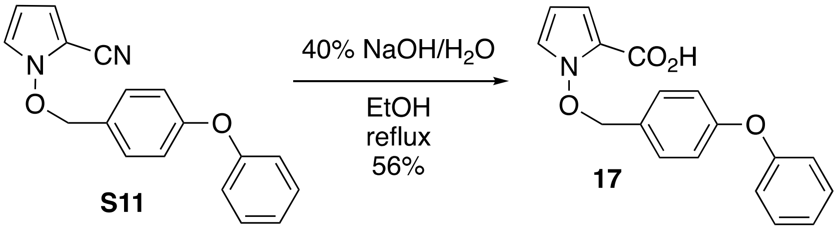

To a solution of 1-((4-phenoxybenzyl)oxy)-1*H*-pyrrole-2-carbonitrile (**S11**) (220 mg, 0.76 mmol) in EtOH (1.6 mL), 40% aqueous NaOH (1.0 mL) was added at room temperature. The reaction mixture was heated at 70 °C for 23 h. After cooling to room temperature, the resulting mixture was

concentrated under reduced pressure. The residue was dissolved in water and pH of the resulting solution was adjusted to 3 by addition of 50% aqueous phosphoric acid. The aqueous layers were extracted with $CHCl_3$ (3×10 mL). The combined organic layers were dried over $MgSO_4$, filtered and concentrated under reduced pressure. The residue was purified by SiO_2 column chromatography (20% EtOAc/*n*-hexanes to 30% EtOAc/*n*-hexanes) to afford 1-((4- phenoxybenzyl)oxy)-1*H*-pyrrole-2-carboxylic acid (**17**, 132 mg, 56%) as orange solid.

^{1}H-NMR (500 MHz, $CDCl_3$) δ 7.39–7.33 (m, 4H), 7.13 (t, *J* = 7.5 Hz, 1H), 7.01 (dd, *J* = 12.3, 8.3 Hz, 4H), 6.93 (dd, *J* = 4.5, 2.0 Hz, 1H), 6.79 (t, *J* = 2.3 Hz, 1H), 5.98 (dd, *J* = 4.5, 3.0 Hz, 1H), 5.21 (s, 2H); ^{13}C-NMR (125 MHz, $CDCl_3$) δ 164.6, 158.5, 156.8, 132.5, 131.8, 130.2, 130.0, 128.6, 124.8, 123.8, 120.4, 119.4, 119.4, 118.7, 117.5, 116.2, 104.7, 81.8; ESI-HRMS $[M+H]^+$: calcd. for $[C_{18}H_{16}NO_4]^+$: 310.1074, found: 310.1044; IR ν_{max} 1680, 1649, 1528, 1508 cm^{-1}; mp 139.4–142.1 °C.
