# Supplementary material for: Discovery, characterization and functional improvement of kumamonamide as a novel plant growth inhibitor that disturbs plant microtubules
Source: Sci Rep. 2021 Mar 23;11:6077. doi: 10.1038/s41598-021-85501-1 (PMC7988157; doi:10.1038/s41598-021-85501-1)
Supplement: Supplementary file 2 — Supplementary Information 2. [file 41598_2021_85501_MOESM2_ESM.pdf]

Discovery, characterization and functional improvement of kumamonamide as a novel plant growth inhibitor that disturbs plant microtubules

Takashi Ishida, Haruna Yoshimura, Masatsugu Takekawa, Takumi Higaki, Takashi Ideue, Masaki Hatano, Masayuki Igarashi, Tokio Tani, Shinichiro Sawa, Hayato Ishikawa

Supporting information Fig. 1

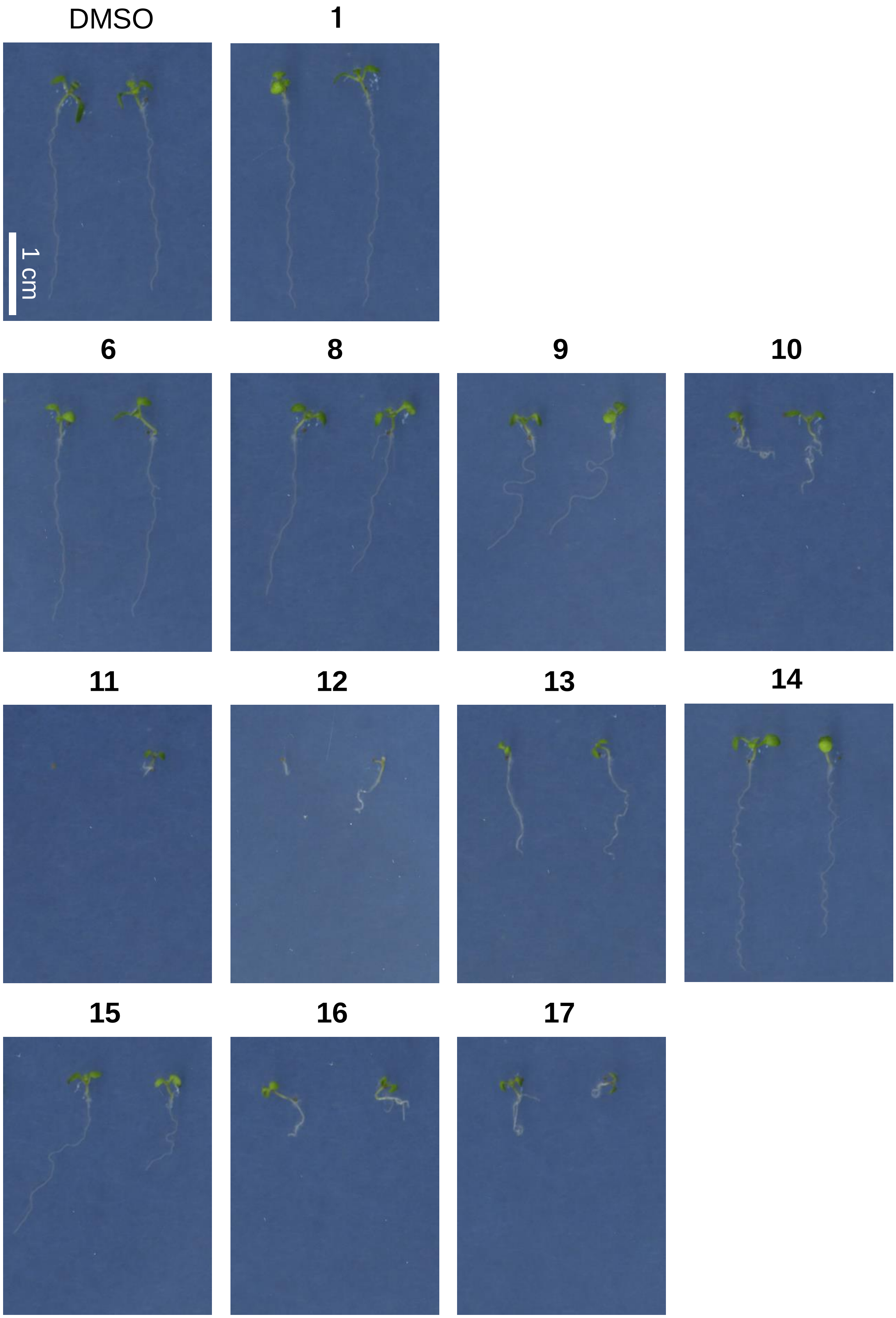

Supporting information Fig. S1
7-day-old seedlings grown on MS media with or without 50 μM kumamonamic acid derivatives.

supporting information Fig. 2

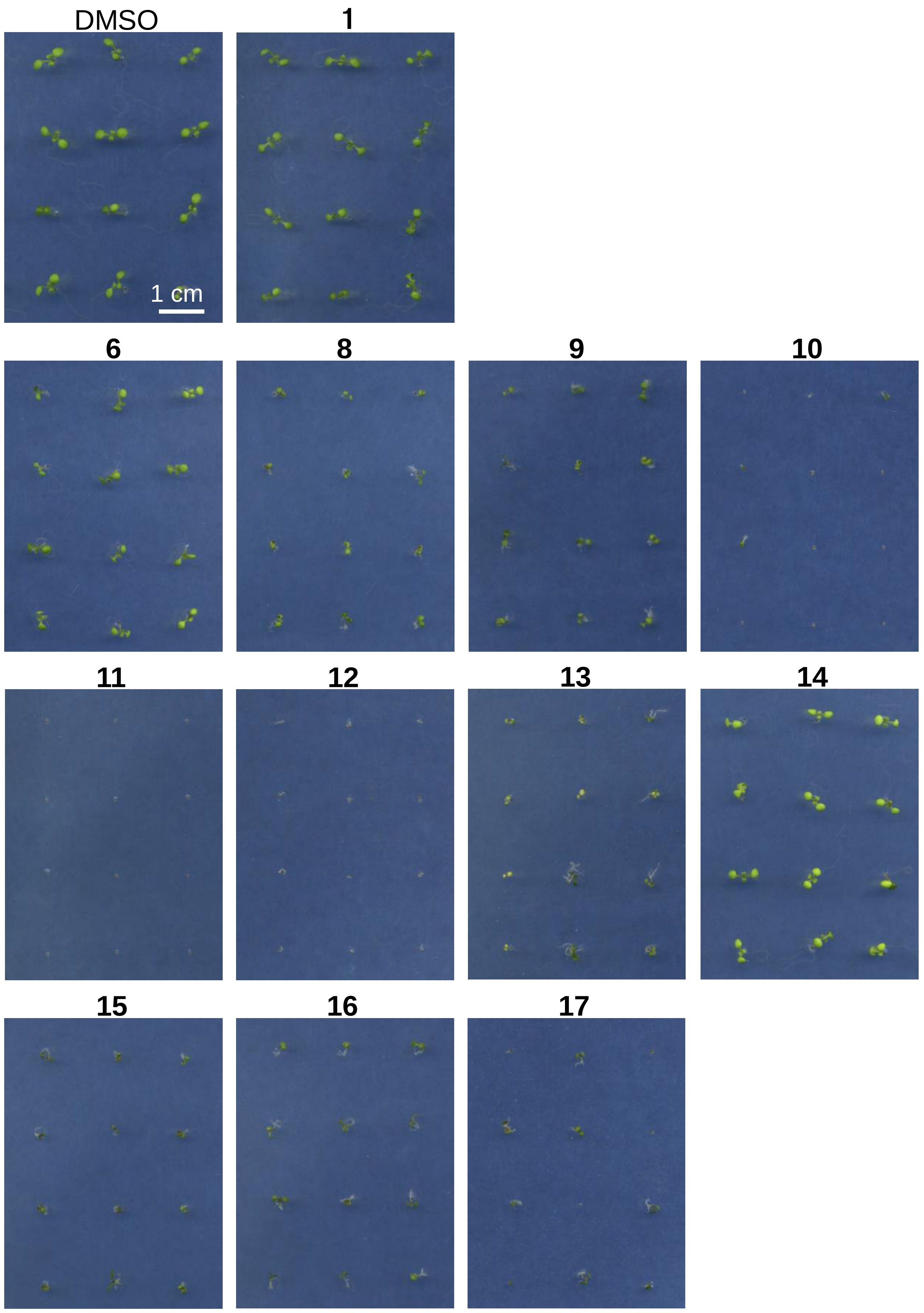

Supporting information Fig. S2
7-day-old seedlings grown on MS media with or without 200 μM kumamonamic acid derivatives.

supporting information Fig. 3

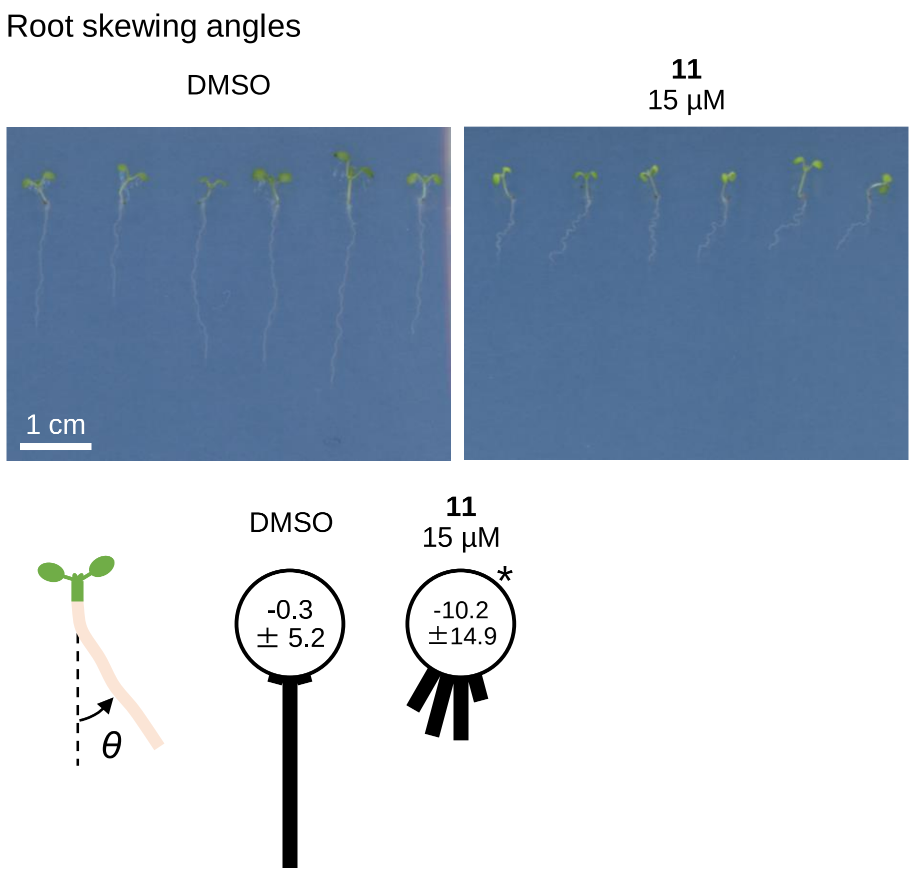

Supporting information Fig. S3
Lower concentration effect of KAND **11** in Arabidopsis roots. Root skewing angles measured in the Mock (DMSO) or 15 µM KAND **11**. This root skewing assay was performed independent to the assay presented in Fig 6. The root growth direction of seedlings grown on DMSO-containing media was not different from the control condition shown in fig 6. Asterisks indicate significant differences from mock treatment (*t*-tests, $p<.0.05$). $n > 22$. Scale bar = 1 cm.

supporting information Fig. 4

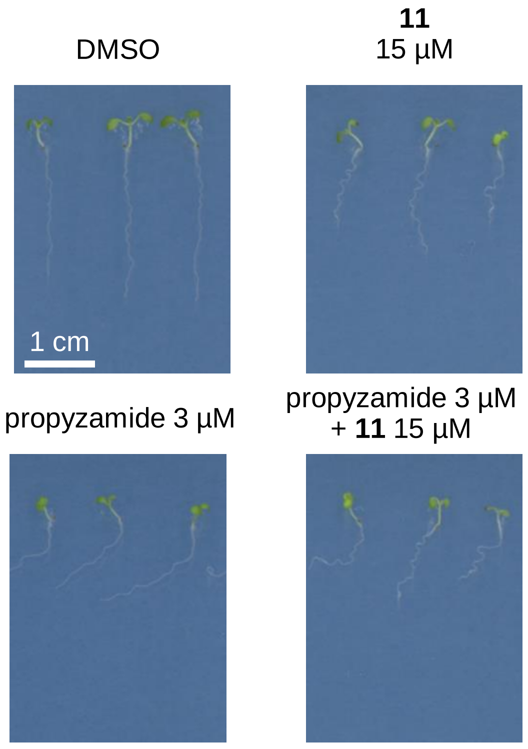

Supporting information Fig. S4
Combined chemical treatment. Arabidopsis seedlings grown on agar medium containing 15 µM KAND **11** and/or 3 µM propyzamide. Scale bar = 1 cm.

supporting information Fig. 5

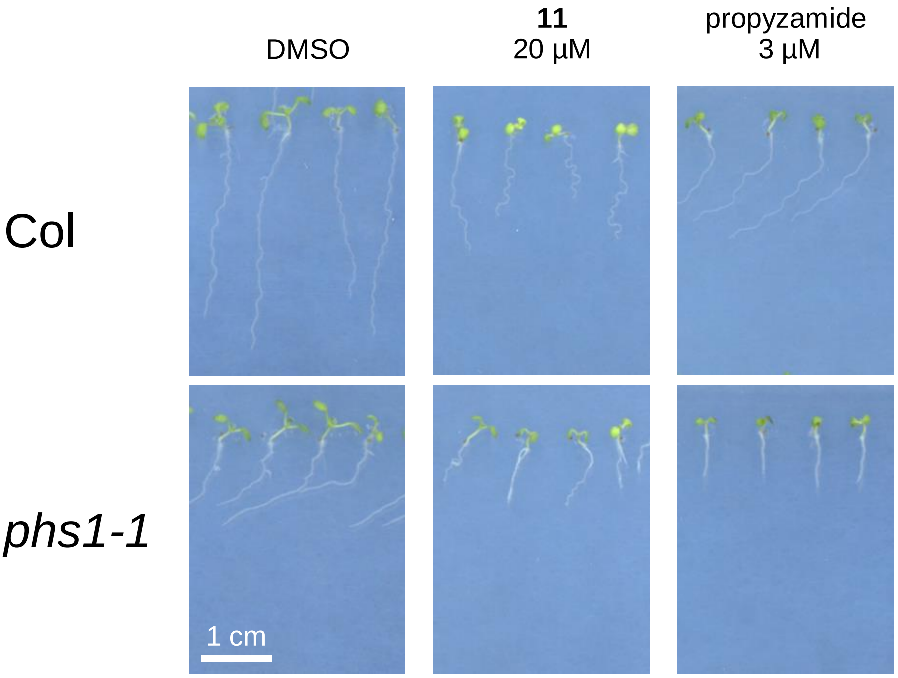

Supporting information Fig. S5
Effect of KAND **11** in microtubule inhibitor hypersensitive mutant. Wild-type (Col) or *propyzamide hypersensitive 1-1* (*phs1-1*) seedlings grown on agar medium containing 15 µM KAND **11** or 3 µM propyzamide. Scale bar = 1 cm.

Supporting information Fig. 6

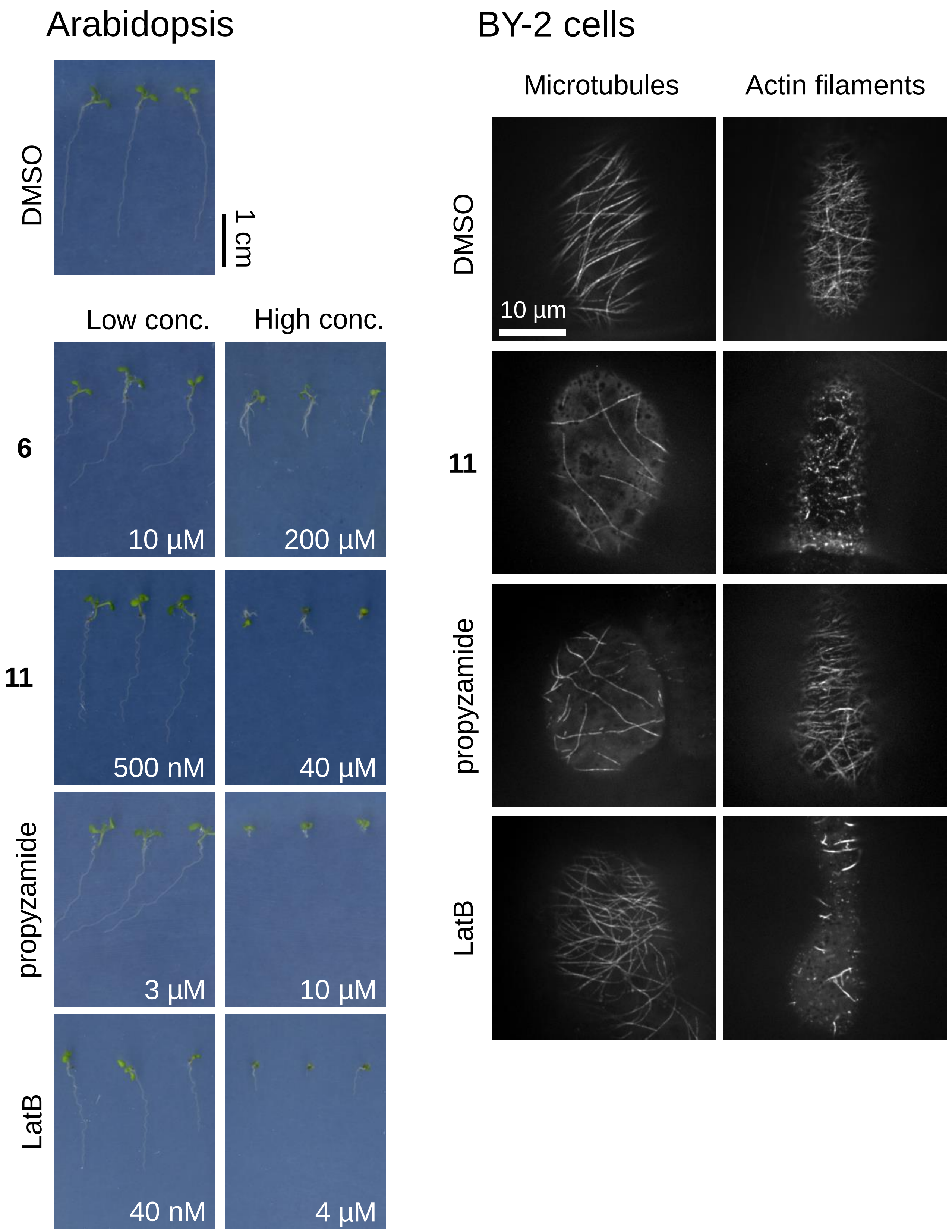

Supporting information Fig. S6
Effect of kumamonamic acid 6, KAND **11**, propyzamide or latlanculin B (LatB) in Arabidopsis seedlings or cytoskeletons of tobacco BY-2 cells.

Supporting information Fig. 7

Immunostaining in HeLa cells β-tublin/DAPI

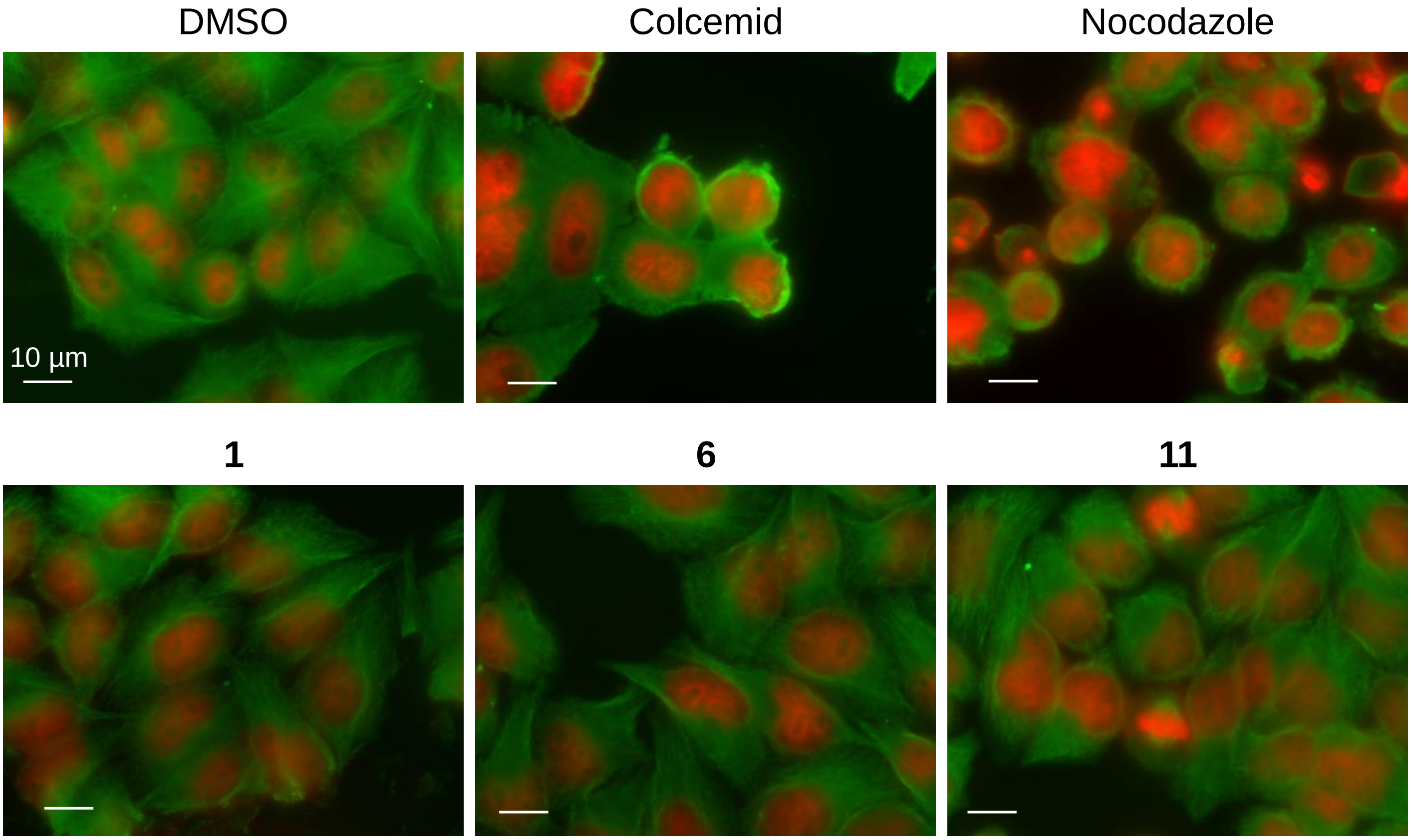

Supporting information Fig. S7
Effect of 100 µM kumamonamide **1**, 100 µM kumamonamic acid **6**, 100 µM KAND **11**, 100 ng/ml colcemid or 100 ng/ml nocodazole in HeLa cells. Microtubules (green) were visualized with anti-tubulin antibody. Nuclei (red) were stained with DAPI. Scale bar = 10 µm.
